# ABAP1 Plays a Role in the Differentiation of Male and Female Gametes in *Arabidopsis thaliana*

**DOI:** 10.3389/fpls.2021.642758

**Published:** 2021-02-10

**Authors:** Luiz M. Cabral, Hana P. Masuda, Helkin F. Ballesteros, Janice de Almeida-Engler, Márcio Alves-Ferreira, Karen L. G. De Toni, Fernanda M. Bizotto, Paulo C. G. Ferreira, Adriana S. Hemerly

**Affiliations:** ^1^Instituto de Bioquímica Médica Leopoldo de Meis, Universidade Federal do Rio de Janeiro, Rio de Janeiro, Brazil; ^2^Departamento de Biologia Celular e Molecular, Instituto de Biologia, Universidade Federal Fluminense, Niterói, Brazil; ^3^Centro de Ciências Naturais e Humanas, Universidade Federal do ABC, São Bernardo do Campo, Brazil; ^4^Institut National de la Recherche Agronomique, Centre National de la Recherche Scientifique, Institut Sophia Agrobiotech, Université Côte d’Azur, Sophia Antipolis, France; ^5^Departamento de Genética, Instituto de Biologia, Universidade Federal do Rio de Janeiro, Rio de Janeiro, Brazil; ^6^Instituto de Pesquisas Jardim Botânico do Rio de Janeiro, Rio de Janeiro, Brazil

**Keywords:** male gametophyte differentiation, female gametophyte differentiation, ABAP1, DNA replication, TCP, ADAP

## Abstract

The correct development of a diploid sporophyte body and a haploid gametophyte relies on a strict coordination between cell divisions in space and time. During plant reproduction, these divisions have to be temporally and spatially coordinated with cell differentiation processes, to ensure a successful fertilization. Armadillo BTB Arabidopsis protein 1 (ABAP1) is a plant exclusive protein that has been previously reported to control proliferative cell divisions during leaf growth in Arabidopsis. Here, we show that ABAP1 binds to different transcription factors that regulate male and female gametophyte differentiation, repressing their target genes expression. During male gametogenesis, the ABAP1-TCP16 complex represses *CDT1b* transcription, and consequently regulates microspore first asymmetric mitosis. In the female gametogenesis, the ABAP1-ADAP complex represses *EDA24-like* transcription, regulating polar nuclei fusion to form the central cell. Therefore, besides its function during vegetative development, this work shows that ABAP1 is also involved in differentiation processes during plant reproduction, by having a dual role in regulating both the first asymmetric cell division of male gametophyte and the cell differentiation (or cell fusion) of female gametophyte.

## Introduction

The life cycle of higher plants alternates between the growth of a diploid sporophytic body and a haploid gametophytic form. The correct development of both generations relies on a strict coordination between cell division and cell differentiation in space and time (Harashima and Schnittger, 2010). The plant body grows mainly as a result of the increase in cell numbers through proliferative and symmetric cell divisions, producing cells of the same size and fate (De Smet and Beeckman, 2011; Rasmussen et al., 2011). In parallel, the formative and asymmetric divisions generate daughter cells of distinct sizes and identities, having an important role in the differentiation of cell types (Kajala et al., 2014).

A proper temporal and spatial coordination between cell division and differentiation during development of both gametophytes is essential for a successful plant reproduction. After meiosis, asymmetric mitotic cell divisions participate in the differentiation of male gametophyte, by forming a large vegetative cell (VC) and a small generative cell (GC) that subsequently divides symmetrically, forming the two sperm cells (Borg and Twell, 2010; Twell, 2011). During female gametogenesis, cell polarity is a key event for the differentiation of the female gametophyte in the mature embryo sac, which is finally composed of seven cells: three antipodal cells at the chalaza; two synergids and the egg cell at the micropyla; and a central cell (Drews and Koltunow, 2011; Schmid et al., 2015). Although the cellular dynamics of male and female gametogenesis are well described, the underlying molecular and biochemical mechanisms temporally coordinating both processes remain to be elucidated.

G1/S transition is a key point of coordination between cell division and cell differentiation homeostasis (Sablowski and Carnier Dornelas, 2014). RETINOBLASTOMA-RELATED 1 (RBR1) is a key regulator of G1/S transition in *Arabidopsis*, having a critical role in integrating cell proliferation and differentiation during *Arabidopsis* sporophyte development (Berckmans and De Veylder, 2009; Harashima and Sugimoto, 2016). During leaf development, RBR1-E2F complex acts as a repressor of cell division in proliferating cells, inhibits the transition into endocycle entry, and prevents ectopic division and recurrent differentiation of guard cells (Park et al., 2005) by repressing the expression of a pre-replication complex (pre-RC) member – CDC6 - and other S-phase genes (Desvoyes et al., 2006). In *Arabidopsis* roots, RBR1 functions through associations with other proteins, by promoting cell differentiation in the root meristem and regulating asymmetric cell division in *Arabidopsis* root stem cell niche (Cruz-Ramírez et al., 2012; Weimer et al., 2012). Besides mediating female germline specification by repressing WUSCHEL (WUS) in megaspore mother cell, RBR1 is also involved in the control of both male and female gametophyte development, as *rbr1-1* mutants showed supernumerary nuclei in the embryo sac and in pollen grains (Ebel et al., 2004; Chen et al., 2009; Zhao et al., 2017).

Armadillo BTB *Arabidopsis* Protein 1 (ABAP1) is another central player at G1/S transition in *Arabidopsis* through its dual role in the regulation of DNA replication and transcription. During *Arabidopsis* vegetative development, ABAP1 acts by balancing rates of proliferative cell divisions during leaf growth, through negatively regulating DNA replication and transcription, in a mechanism that controls the DNA replication factor CDT1a/b availability and consequently, the assembly of pre-RC (Masuda et al., 2008). In this work, we assessed ABAP1’s role during plant reproduction. We showed that *ABAP1* is expressed in both male and female gametophytes and that ABAP1 imbalance impairs both male and female gametogenesis. The fundamental molecular and biochemical mechanisms of action of ABAP1 were determined in the gametophytic development context. Protein pull down, Electrophoretic Mobility Shift Assay (EMSA) and chromatin immunoprecipitation (ChIP) assays revealed that ABAP1 binds to the transcription factors TCP16 (a class I TCP) and ARIA-interacting Double AP2-domain (ADAP, a member of AINTEGUMENTA family), repressing their target genes expression in the male and female gametogenesis, respectively. The ABAP1-TCP16 complex binds to the *CDT1b* promoter to repress its transcription and consequently regulate microspore first asymmetric cell division in the male gametophyte. Besides, the ABAP1-ADAP complex binds to the *Embryo sac Development Arrest 24-like* (*EDA24-like*) promoter, repressing its transcription and regulating polar nuclei fusion to form the central cell in the female gametophyte. Therefore, ABAP1 is involved in the regulation of both formative cell division in male gametophyte and cell differentiation (or cell fusion) in female gametophyte, two processes that are essential for a successful plant reproduction in *Arabidopsis*.

## Materials and Methods

### Plant Material and Growth Conditions

Columbia-0 (Col-0), Landsberg *erecta* (L*er*), *cdt1b*^*KD*^ (SALK_001276), and *eda24-like^*KD*^* (SALK_121133) were obtained from *Arabidopsis* Biological Resource Center (ABRC). ABAP1^OE^, *abap1*^*ET*^ and ABAP1Pro:GUS lines were previously described (Masuda et al., 2008). Homozygous SALK lines were confirmed by PCR genotyping (primers are available in Supplementary Information).

Seeds were surface sterilized (70% ethanol for 2 min, 5% bleach for 10 min, dH_2_O washed for five times), plated in Murashige and Skoog agar plates [4.43 g L^–1^, 0.8% agar (w/v), 1% sucrose (w/v), 0.5 g L^–1^ MES] and kept at 4°C for 2 days prior to transfer to a growth room at 23°C with a 16 h light/8 h dark cycle. After 7 days, *in vitro* plants were transferred to a mixture of soil:vermiculite (2:1) and grown under the same conditions as described above.

### Constructs

The full-length coding regions of *ABAP1, TCP24, CDT1a, CDT1b, TCP16* and *ADAP* were PCR amplified with the specific primers as described in Supplementary Information and cloned into entry vectors pDONR201 or pDONR221. The entry clones were then recombined with destination vectors (pDEST15 and pDEST17 for protein expression in *Escherichia coli*; pDESTDBD and pDESTAD, for yeast two hybrid experiments). Vectors for ABAP1 and TCP24 overexpression in plants are described elsewhere (Masuda et al., 2008). All constructs were made using Gateway Technology (Invitrogen) except for *in situ* hybridization, where 1709 bp of *CDT1a* and 1461 *CDT1b* CDS fragments were cloned into pGEMT Easy Vector (Promega).

### Microscopic Analysis

Plant material was harvested at the indicated developmental stages and fixed with 4% paraformaldehyde in 100 mM sodium phosphate buffer (pH 7.2). Fixed material was processed for historesin or paraplast infiltration and then sectioned; or cleared for DIC microscopy observation. Details of microscopy analyses are available in the Supplementary Information.

### Promoter GUS Experiments and *in situ* Hybridization

Flowers of 6-weeks-old homozygous ABAP1Pro:GUS plants grown in the soil were used for histochemical localization of GUS activity. GUS stained material was observed under light or DIC microscope (Axiophot, Zeiss). For more details, see Supplementary Information.

For *in situ* hybridization, open flowers and flower buds of 6-week-old plants were fixed and hybridized essentially as described previously (de Almeida Engler et al., 2001). Gene-specific sense and antisense probes of *CDT1a* and *CDT1b* genes were labeled using PCR DIG Probe Synthesis Kit (Roche). Probes were detected using an anti-digoxigenin antibody to which alkaline phosphatase had been conjugated (Roche Diagnostics). Images were analyzed in a stereomicroscope.

### Quantitative Real Time PCR (qRT-PCR)

RNA extraction, reverse transcription and semi quantitative real time PCR were performed as described previously with minor modifications (Masuda et al., 2004). Total RNA was extracted from frozen materials (Logemann et al., 1987), treated with RNAse-free Ambion^TM^ DNAse I and first strand cDNA was synthesized using “High Capacity cDNA Reverse Transcription Kit” according to the manufacturer’s instructions (Thermo Fisher Scientific). For qPCR, cDNA was amplified using SYBR-Green^®^ PCR Master kit (Perkin-Elmer Applied Biosystem) on the GeneAmp 9600 thermocycler (Perkin-Elmer Applied Biosystems) under standard conditions. Relative expression was calculated using 2^−ΔΔCT^ method with *UBQ14* as constitutive gene. qPCR values are means from three technical replicates and at least two biological replicates. Primer sequences for all genes used in qPCR are listed in Supplementary Information.

### Microarray Experiment

Microarrays were based on the *Arabidopsis* Genome Oligo Set version 1.0 (Operon), manufactured as previously described (Wellmer et al., 2004) and were kindly donated by Dr. Elliot Meyerowitz (Caltech, United States). Plant material, RNA extraction, antisense RNA labeling, raw data processing and data analyses are described in Supplementary Information. Microarray data was deposited in Gene Expression Omnibus (no. GEO: GSE164480, GSM5011985).

### Yeast Two-Hybrid Assay

Yeast two hybrid assay was carried out essentially as described by Masuda et al. (2008). Pairs of interacting proteins were cloned in both AD and DBD vectors and assayed for yeast two hybrid to reduce false positive results. For details, see Supplementary Information.

### *In vitro* Protein Interaction Assay (GST-Pull Down)

ABAP1-GST, AtTPC24-GST, TCP16-HIS and ADAP-HIS were expressed in *E. coli* strain BL21 as described previously (Chekanova et al., 2000), with modification in the lysis buffer [25 mM Tris, pH 8.0, 1 mM EDTA, 10% glycerol, 50 mM NaCl, 0.1% Triton X-100, 1 mM phenylmethylsulfonyl fluoride (PMSF), 10 mM leupeptin, and 75 mM aprotinin]. GST-pull down analyses were carried following a protocol described elsewhere (Tarun and Sachs, 1996). For details, see Supplementary Information.

### Electrophoretic Mobility Shift Assay (EMSA)

Electrophoretic mobility shift assays were performed essentially as described previously (Masuda et al., 2008). Details on experimental procedure and sequences probes are available in Supplementary Information.

### Chromatin Immunoprecipitation (ChIP), PCR Amplification and qRT-PCR Experiment

Flower buds of wild-type (Col-0) and ABAP1^OE^ were collected, fixed in 1% formaldehyde under vacuum for 15 min followed by 5 min incubation in 100 mM glycine to stop the crosslinking, frozen in liquid nitrogen and then stored at −80°C. Chromatin isolation and immunoprecipitation was carried out as described before (Gendrel et al., 2005) with minor modifications (an extra 5 min wash was added at each washing step). Anti-ABAP1 polyclonal antibody described elsewhere (Masuda et al., 2008) was used in the IP step. ABAP1-IP gDNA, input gDNA and mock-IP gDNA were used in PCR amplification using primers specific to promoter and coding regions of *EDA24-like*, *CDT1a* and *CDT1b*. Whole cell extract, immunoprecipitated WT and immunoprecipitated ABAP1^OE^ materials were used in qRT-PCR. For ChIP-qPCR, ChIP DNA was analyzed by qPCR using the indicated primer pairs (sequences are available in Supplementary Information). Relative enrichment for each fragment was calculated first by normalizing the amount of the amplified DNA fragment with the Actin 2 fragment, and then the ratio of ABAP1^OE^/wild-type was calculated using the following equation: 2^(CtABAP1OE Actin^ ^2–CtABAP1OE ChIP fragment)^/2^(CtWT Actin^ ^2–CtWT ChIP fragment)^. Each PCR was repeated three times, the mean value of technical replicates was recorded for each biological replicate and error bars represent the SD from three independent experiments.

### Accession Numbers

Sequence data from this article can be found at the *Arabidopsis* Genome Initiative database with the following accession numbers: ABAP1 (At5g13060), Actin 2 (At3g18780), UBQ14 (AT4G02890), CDT1a (At2g31270), CDT1b (At3g54710), TCP16 (AT3G45150), EDA24-like (At1g23350), and ADAP/WRI3 (At1g16060).

## Results

### Deregulation of ABAP1 Expression Affects Plant Reproduction

To assess if ABAP1 has a role during plant reproduction processes, plants with increased or reduced expression levels of ABAP1 were characterized. The studies were performed with *Arabidopsis* plants expressing 5- to 17-fold and 2- to 5-fold higher levels of *ABAP1* mRNA and protein, respectively (ABAP1^OE^) and a heterozygous enhancer trap line (*abap1*^*ET*^) showing a two and five fold reduction in *ABAP1* mRNA and protein levels, respectively (Masuda et al., 2008).

**FIGURE 1:**
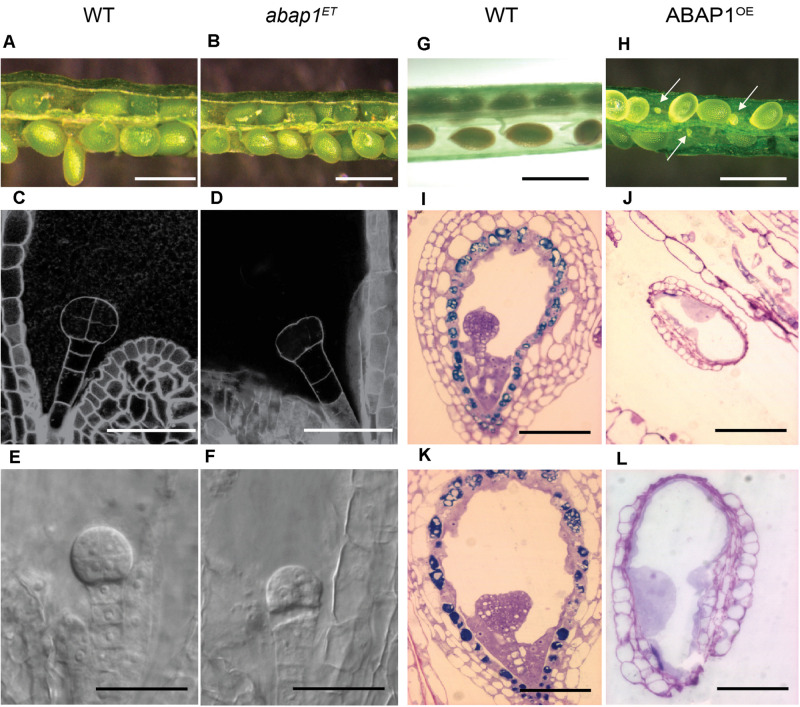
Embryo development in plants with altered levels of ABAP1. Images of siliques at the same developmental stages, comparing embryo and seed development in wild-type (WT) and mutant plants. Left panels **(A–F)** shows comparison between WT and *abap1*^*ET*^ plants. **(A,B)** Open siliques of WT and *abap1*^*ET*^ plants showing normal seed size. **(C–F)** Initial events in embryo development from WT and *abap1*^*ET*^ seeds. Dark field illumination microscopy of WT seeds with embryo at 8 cell stage **(C)**, and *abap1*^*ET*^ seeds with the embryo at the initial zygotic divisions **(D)**. Nomarski microscopy of embryos from WT plants at globular cell stage **(E)** and from *abap1*^*ET*^ plants with defects after the initial zygotic divisions **(F)**. Right panels **(G–L)** shows comparisons between WT and ABAP1^OE^ plants. **(G)** Open WT siliques with normal developing seeds; and **(H)** open ABAP1^OE^ siliques with small and shriveled seeds (arrows). **(I,K)** Micrographs of sections of WT seeds with embryos at globular **(I)** and transition to early heart stages **(K)**. **(J,L)** Micrographs of sections of ABAP1^OE^ seeds that did not develop and/or arrested before fertilization **(J)**. **(L)** is a higher magnification of **(J)**. Scale bars: 1 mm in **(A,B,G,H)**; 20 μm in **(C–F)**; 100 μm in **(I–K)**; and 50 μm in **(L)**. For *abap1*^*ET*^ and WT Landsberg *erecta* background plants, a total of 300 seeds in 15 siliques were analyzed for each genotype. For ABAP1^OE^ a total of 708 seeds were analyzed in 36 siliques, and for Col-0 WT plants 432 seeds were analyzed in 18 siliques.

Homozygous plants could not be rescued in either the *abap1*^*ET*^ or in the ABAP1^OE^ lines, suggesting that extreme up or down-regulation of *ABAP1* can cause a lethal phenotype. Microscopic analyses of seed development showed that embryogenesis was arrested at pre-globular stage in 32% of the *abap1*^*ET*^ seeds (compare Figures 1C,E with 1D,F). Nevertheless, at later stage (mature green), seeds formed seed coat, and no difference in size and color was observed among the seeds in a complete silique (Figure 1B) when compared to the wild-type silique (Figure 1A). Germination frequency was quantified and 32% of *abap1*^*ET*^ seeds did not germinate, in correspondence with the frequency of arrested embryos observed earlier after fertilization.

ABAP1^OE^ plants, however, produced siliques shorter than those in wild- type plants and exhibited an average of 53–62% undeveloped, very small and white colored seeds compared to wild-type plant seeds (Figures 1G,H). The ABAP1^OE^ mutant phenotype was different from the abortion seed phenotype observed in *orc2* (Collinge et al., 2004), *ttn4* (Tzafrir et al., 2002), and *raspberry* (Yadegari et al., 1994) mutants. Microscopic analyses of ABAP1^OE^ siliques indicated that embryogenesis was not seen in the undeveloped seeds, suggesting that either gametogenesis, fertilization or zygote activation was impaired (Figures 1I–L). Abnormal structures within the embryo sac, that could suggest formation of endosperm, were observed in ABAP1^OE^ seeds.

**FIGURE 2:**
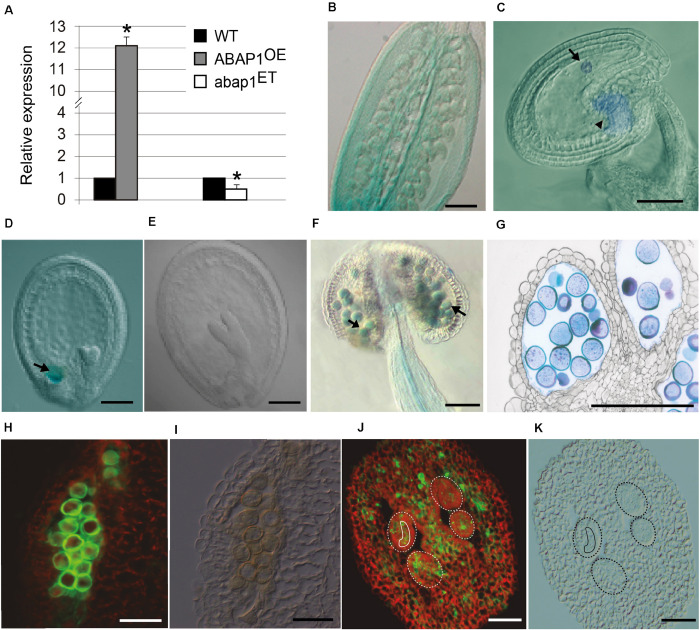
ABAP1 expression in flowers. **(A)** Relative mRNA levels of ABAP1 were determined by qRT-PCR in wild-type, ABAP1^OE^ and abap1^ET^ flowers. Data were normalized with UBQ14 as reference gene and were compared with wild-type. Data shown represent mean values obtained from independent amplification reactions (*n* = 3) and biological replicates (*n* = 2). Each biological replicate was performed with a pool of 15 inflorescences. Bars indicate mean ± standard error of biological replicates. A statistical analysis was performed by *t*-test (*p*-value < 0.05). Asterisks (*) indicate significant changes. **(B–G)** Nomarski microscopy of cleared tissues. **(B)** promABAP1:GUS activity in the ovary prior to fertilization. **(C)** promABAP1:GUS activity early after fertilization. Arrow and arrowhead point to embryo and chalaza expression, respectively. **(D)** Decrease in GUS activity in embryos of 72 h (heart stage). The expression in the chalazal chamber, however, remains (arrow). **(E)** In torpedo stage, GUS activity is not seen neither in the embryo or chalaza. **(F)** promABAP1:GUS activity in anthers. Arrow points to GUS activity in pollen. **(G)** Cross section of anthers with promABAP1:GUS activity in pollen. **(H,J)** Localization of ABAP1 protein by epifluorescence of reproductive tissues using anti-ABAP1 antibodies conjugated with Alexa Fluor 488 and **(I,K)** corresponding DIC microscopy. **(H,I)** ABAP1 protein localization in a pollen sac cross section. **(J,K)** ABAP1 protein localization in cross section of ovary and ovules. Dashed lines indicate the perimeter of ovules and the full line is around the embryo sac. The scale bars correspond to 100 μm in **(B)**, 20 μm in **(C–G)**, and to 10 μm in **(H–K)**.

Previous studies on *ABAP1* gene expression have shown high GUS activity in developing flower buds, with strong expression in the ovary and petals (Masuda et al., 2008). qRT-PCR was used to confirm that *ABAP1* mRNA levels were altered in mutant flower buds, showing a 12-fold increase in ABAP1^OE^ and a two-fold reduction in *abap1*^*ET*^ lines (Figure 2A). To determine the spatial localization of *ABAP1* expression in *Arabidopsis* gametogenesis, ABAP1Pro:GUS plants were analyzed. GUS activity was detected overall the ovary (Figure 2B). A time-lapse investigation of GUS activity indicated that *ABAP1* expression remained in the chalaza and in the embryo very early after fertilization (Figure 2C), decreased at mid-heart stage (Figure 2D) and disappeared only during the torpedo stage of embryo development (Figure 2E).

During male gametogenesis, GUS assays indicated that *ABAP1* is expressed in pollen and is regulated according to its developmental stage, since it appears only after microsporogenesis. Thus, ABAP1 is present during pollen differentiation, around stage 10–11 of anther development (Figures 2F,G), when the first mitotic asymmetric cell division occurs (Sanders et al., 1999).

ABAP1 expression in wild-type was also confirmed by immunolocalization in pollen (Figures 2H,I) and ovary, including the ovules and embryo sac (Figures 2J,K) by using anti-ABAP1 antibodies conjugated with Alexa Fluor 488. During male gametogenesis, immunolocalization experiments suggest that ABAP1 is initially present at low levels in the tetrads. As gametogenesis proceeds, higher levels of ABAP1 are observed in the vacuolate stage of microspore, before the first mitotic division. Then, its levels decrease at subsequent stages of gametogenesis (Supplementary Figure 1). Immunolocalization of ABAP1 protein was also analyzed in ABAP1^OE^ plants (Supplementary Figure 2) confirming that plants overexpressing *ABAP1* under the control of the 35S promoter have a higher fluorescence signal in the reproductive organs, including the male and female gametophytes (Supplementary Figures 2C–H), when compared to the wild-type plant (Supplementary Figures 2A–F). Immunofluorescence controls without anti-ABAP1 antibody or using pre-immune serum did not show fluorescent signal (Supplementary Figures 2I–P).

Altogether, the data showed that ABAP1 has a very specific spatial and temporal expression during the gametophytic phase and that its deregulation affects plant reproduction. Down regulation of ABAP1 disturbed initial mitotic cell divisions in the embryo formation after fertilization, possibly due to its essential role in DNA replication and mitotic cell division. On the other hand, plants with higher levels of ABAP1 lacked developing embryos in the shriveled seeds, suggesting that either fertilization or zygote activation is defective in ABAP1^OE^. Because ABAP1 is a transcriptional repressor, higher levels of ABAP1 could potentially enhance the repression of important genes for these processes or in the gametogenesis, representing a novel role other than the one in proliferative cell division.

### Plants With Ectopic Expression of ABAP1 Have Defects in Pollen Development

To establish the basis for the reduced fertility of ABAP1^OE^ plants, we analyzed whether higher levels of ABAP1 interfered in the different processes of flower development. Defects were found exclusively in male and female gametophytes development.

Optical microscopic analyses were performed at different stages of the male gametophyte development of ABAP1^OE^ and of the wild-type plants (Supplementary Figure 3). The first phenotypic differences were noticed after meiosis, at stage 11 of flower development, when the cytoplasm of some pollen grains from ABAP1^OE^ flowers retracted (Supplementary Figure 3D). At this stage, the first asymmetric mitotic division (Pollen Mitosis I, PMI) is supposed to happen. At stage 12, when the bilocular anther is formed and the pollen grains are mature, 45% of pollen grains were malformed and shriveled in the anthers of the ABAP1^OE^ flowers (Supplementary Figure 3E).

**FIGURE 3:**
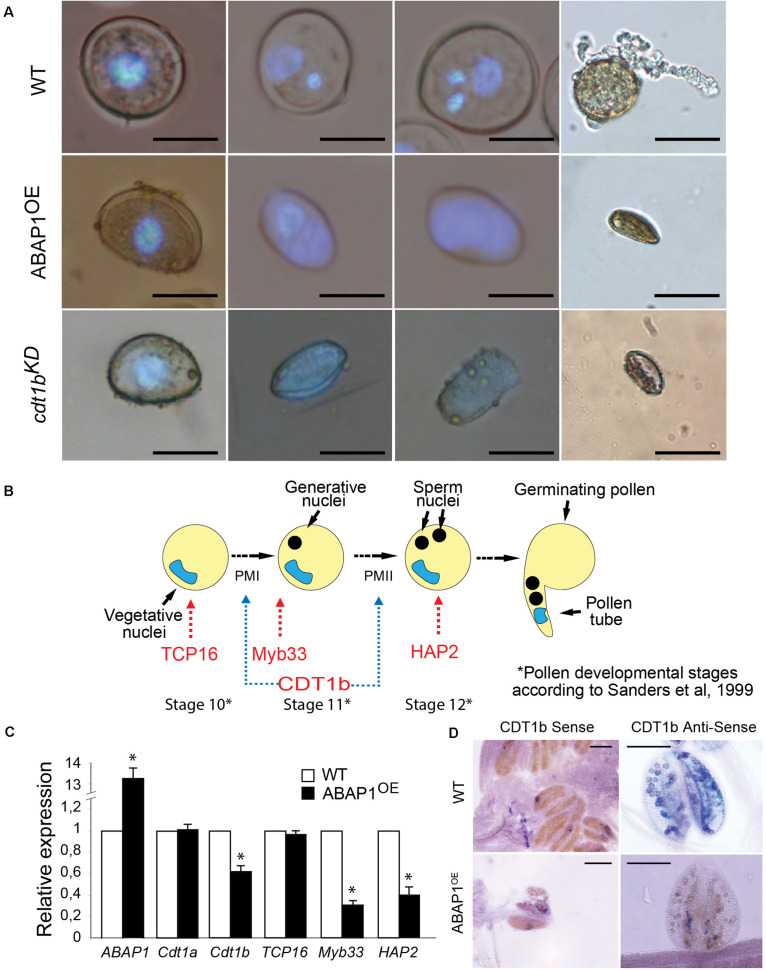
Defective pollen maturation in ABAP1^OE^ plants. **(A)** Pollen grains stained with DAPI in different developmental stages, in WT plants (upper line), ABAP1^OE^ plants (middle line) and *cdt1b*^*KD*^ (bottom line). **(B)** Schematic representation of the series of events taking place after meiosis, during the maturation of the male gametophyte in Arabidopsis. PMI and PMII are the two mitotic divisions necessary for the male gametophyte development. Red arrows indicate marker genes of pollen developmental stages. Blue arrows indicate the stages when CDT1b might be required for mitotic cell division. **(C)** Relative mRNA levels of *ABAP1, CDT1a, CDT1b, TCP16, Myb33*, and *HAP2* were determined by qRT-PCR in wild-type and ABAP1^OE^ anthers at stage 12 of development (according to Sanders et al., 1999). Data were normalized with *UBI14* as reference gene and were compared with wild-type. Each biological replicate was performed with a pool of 50 anthers. Bars indicate mean ± standard error of biological replicates. A statistical analysis was performed by *t*-test (*p*-value < 0.05). Asterisks (^∗^) indicate significant changes. **(D)** *CDT1b* mRNA levels in wild-type and ABAP1^OE^ anthers using whole mount *in situ* hybridization with anti-sense probes. Hybridization signals are seen as purple dots under bright field optics. The scale bars represent 20 μm in **(A)** and 0.2 mm in **(D)**.

To determine in which stage of male gametogenesis ABAP1^OE^ was affected, nuclei of microspores at different developmental stages of gametogenesis were visualized by DAPI staining. The anthers were collected at stages 10, 11, and 12 of flower development (Smyth et al., 1990), which correspond to male gametophytes with one, two, and three nuclei in wild-type plants, respectively (Figure 3A, upper line and Figure 3B). Male gametophyte of plants with higher levels of ABAP1 presented only one diffuse nucleus, suggesting that they were arrested before Pollen Mitosis I (PMI), the asymmetric cell division that forms the vegetative and generative cells (Figure 3A, middle line). This phenotype resembles the one observed in *cdt1b*^*KD*^ mutants (Figure 3A, bottom line), a pre-RC component and target of ABAP1 regulation mediated by TCP24 (Masuda et al., 2008). Pollen germination assays showed that normal shaped pollen grains from wild-type flowers germinated normally (Figure 3A, upper line), while shriveled pollen from ABAP1^OE^ anthers could not germinate (Figure 3A, middle line). These results suggest that ABAP1 may regulate male gametophyte development at PMI.

### ABAP1 Interacts With TCP16 and Regulates *CDT1b* Levels in the Male Gametophyte

In leaves, ABAP1 negatively regulates mitotic cell divisions by repressing *CDT1a* and *CDT1b* expression, in an association with the transcription factor TCP24 (Masuda et al., 2008). To determine the molecular mechanisms behind ABAP1’s effects on the first mitotic division of pollen development, a mechanism similar to the one regulating mitotic proliferative cell divisions was tested.

**FIGURE 4:**
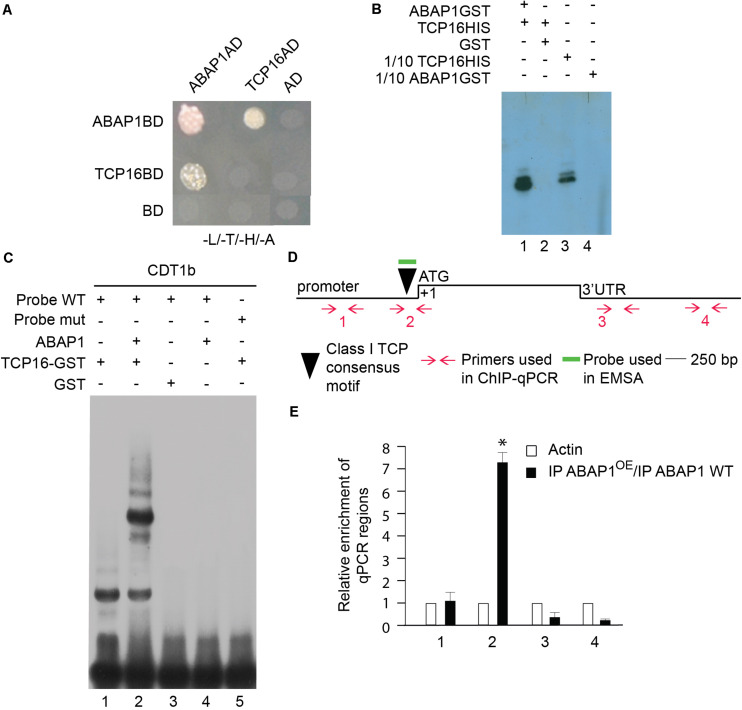
Characterization of the interaction between ABAP1 and TCP16 and their role in transcription. **(A)** Yeast two hybrid interaction assays between ABAP1 and cloned in both AD and BD vectors. Negative controls used DAD and DBD empty vectors. The panel shows conjugated yeasts growing in SD medium lacking leucine, tryptophan, histidine and adenine, for selection of strong protein interactions. **(B)** GST-pull down assay between ABAP1–GST and TCP16-HIS produced in *E. coli*. Western blot analysis used anti-HIS antibody. Lane 1, TCP16-HIS with ABAP1-GST; lane 2, TCP16-HIS with GST alone; lanes 3 and 4, 1/10 inputs of ABAP1-GST and TCP16-HIS, respectively. **(C)** EMSA of ABAP1, TCP16 and the complex TCP16–ABAP1 with wild-type (WT) and mutated (mut) radiolabeled probes of *CDT1b* promoter regions harboring TCP recognition box. Lane 1, TCP16 binds to *CDT1b* WT probe; lane 2, addition of ABAP1 causes a supershift in the TCP16 binding to *CDT1b* WT probe; GST alone (lane 3) or ABAP1 alone (lane 4) do not bind to *CDT1b* WT probe; lane 5, TCP16 does not bind to *CDT1b* mutated probe. **(D,E)** Chromatin immunoprecipitation of wild-type Col (WT) and ABAP1^OE^ flower buds with anti-ABAP1. **(D)** Schematic representation of the amplified regions of *CDT1b*. Black triangle indicates the class I TCP motif, located at position –130 to –136 bp. Red arrows and numbers indicate primers used for ChIP-qPCR assays, and short green line indicates DNA probe used for EMSA. The translational start site (ATG) is shown at position +1. **(E)** Chromatin immunoprecipitated with anti-ABAP1 antibody was analyzed by qPCR using gene-specific primer sets, numbered as indicated in **(D)**. The graph shows the fold enrichment of qPCR amplified regions at the promoter (*1, 2*), and 3’UTR (*3,4*) of *CDT1b*. The fold enrichment of each amplified region was calculated as a ratio between IP ABAP1^OE^ and IP WT of values normalized with values of the *ACTIN 2* promoter. Error bars represent the SD of three biological replicates. Asterisk (^∗^) indicates significant difference from the enrichment according to Student’s *t* test (*p*-value < 0.05). IP, immunoprecipitated with anti-ABAP1 antibody.

The TCP24 transcription factor is not significantly expressed during male gametogenesis, as determined in public microarray datasets; therefore, another TCP partner was searched. TCP16 acts regulating early pollen development, since reduced levels of TCP16 prevented PMI to occur, in a similar phenotype as the one observed in ABAP1^OE^ pollen (Takeda et al., 2006). TCP16 is highly expressed at the polarized microspore stage (Takeda et al., 2006) similar to what was observed to ABAP1 by immunolocalization experiments (Supplementary Figures 1C,D). Therefore, the association of TCP16 with ABAP1 was tested in yeast two-hybrid experiments that showed strong interaction between the two proteins (Figure 4A). The formation of ABAP1-TCP16 complex was further confirmed *in vitro*, in a GST-pull down assay with ABAP1-GST and TCP16-HIS in which TCP16 bound to ABAP1 (Figure 4B, lane 1) but not to GST alone (Figure 4B, lane 2). The ABAP1 interaction with TCP16 in Arabidopsis flowers was demonstrated in a semi *in vivo* HIS-pull down experiment, where HIS-fused TCP16 was added to protein extracts of *Arabidopsis* flowers buds, purified in a nickel column and tested for the presence of ABAP1 with anti-ABAP1 antibody (Supplementary Figure 4A, lane 4).

Next, mRNA levels of *CDT1a* and *CDT1b*, two pre-RC genes whose expression levels are regulated by ABAP1-TCP24 in leaves, were investigated by qRT-PCR in mature anthers. *TCP16* and two pollen developmental markers of pollen mitosis, *Myb33* (Plackett et al., 2011) and *HAP2* (von Besser et al., 2006), were also included in the analysis (Figures 3B,C). The results showed that in ABAP1^OE^ anthers, the expression level of *TCP16* and *CDT1a* were normal, while the levels of *CDT1b, Myb33* and *HAP2* were significantly reduced (Figure 3C). The reduction of *CDT1b* mRNA levels in pollen of ABAP1^OE^ compared to levels in control plants was also confirmed by whole mount *in situ* hybridization assays in anthers (Figure 3D). The expression data corroborates the cellular analysis, supporting that the male gametophyte was arrested before PMI in ABAP1^OE^ plants, as *CDT1b, Myb33*, and *HAP2* were down regulated, and that TCP16 might participate in this control since its expression level is normal.

Remarkably, a consensus sequence for class I TCPs interaction (TGGGNCC) (Viola et al., 2011) is found in the promoter region of *CDT1b* (TGGGCCC position −130 to −136 bp), but not in *CDT1a* that harbors only a non-canonical TCP interaction sequence (TGGGCAC position −1,335 to −1,342 bp) (Figure 4D and Supplementary Figure 5). To address the possible cooperation of ABAP1 and TCP16 in the control of *CDT1b* gene transcription, the association between both proteins with *CDT1b* promoter was characterized. The ability of the ABAP1–TCP16 heterodimer to recognize the promoter regions of *CDT1b* was confirmed by electrophoretic mobility shift assay (EMSA) performed with probes containing the class I TCP consensus motif of *CDT1b* (Supplementary Information). TCP16 alone and TCP16–ABAP1 were associated with the wild-type probes (Figure 4C, lanes 1 and 2) but TCP16 alone did not associate with the mutated probe (Figure 4C, lane 5). Neither ABAP1 alone, nor GST associated with the wild-type probe (Figure 4C, lanes 3 and 4). The ABAP1-TCP16 binding to *CDT1b*, but not to *CDT1a* promoter, was confirmed *in vivo* in chromatin immunoprecipitation (ChIP) experiments of chromatin extracts of *Arabidopsis* flower buds, using anti-ABAP1 antibody (Supplementary Figure 5). The results revealed that ABAP1 was associated with regions of *CDT1b* promoter containing the class I TCP consensus motif (Supplementary Figure 5B), but not with *CDT1a* promoter (Supplementary Figure 5A). This interaction was detected in wild-type plants (Supplementary Figure 5B2, lane 3). Higher levels of association were consistently detected in ABAP1^OE^ flower buds when compared to wild-type. The immunoprecipitated material was further amplified by qPCR with four different primer pairs specifically designed to hybridize along the *CDT1b* promoter and 3′UTR regions (Figure 4D). Amplification of IP DNA was significantly higher in the qPCR reactions using the pair of primers that hybridize closer to the class I TCP consensus motif. Amplification dropped as the distance to the TCP motif increased (Figure 4E).

Altogether, the data suggests that up-regulation of ABAP1 levels affects the progression of the first asymmetric cell division during male gametophyte development by associating with TCP16 and repressing *CDT1b* expression in pollen. This mechanism of action was further supported by analyses of pollen development in a *CDT1b* T-DNA insertion line (hereafter referred to as *cdt1b*^*KD*^). *cdt1b*^*KD*^ showed a reduction of around 55% of *CDT1b* expression in anthers when compared to wild-type plants (Supplementary Figure 4B) and presented a similar phenotype as the one observed for ABAP1^OE^ plants, with several shriveled pollen that were unable to germinate and could not undergo the first mitotic asymmetric division (Figure 3A, bottom line).

### Plants With Ectopic Expression of ABAP1 Show Defects in Embryo Sac Development

**FIGURE 5:**
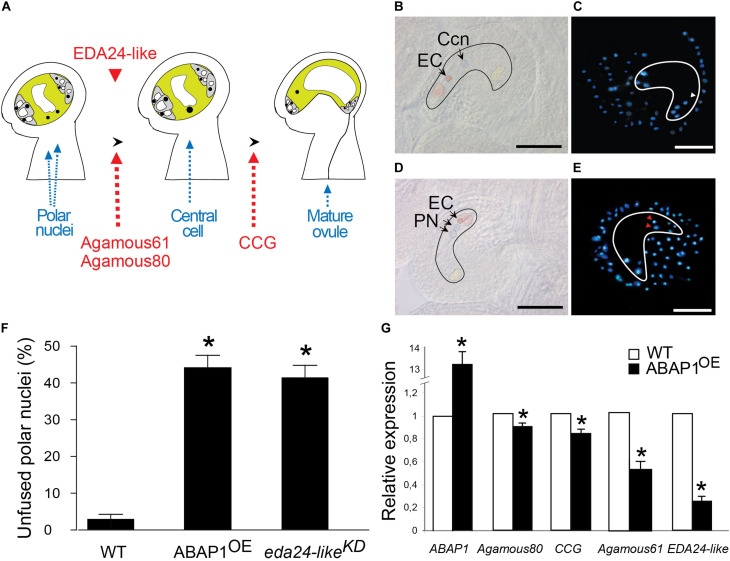
Defective female gametophyte development in ABAP1^OE^. **(A)** Schematic representation of the events taking place during maturation of the female gametophyte. The final embryo sac is a polarized structure with two main distinct regions: the micropyla and the chalaza. Red arrows indicate gene markers of embryo sac developmental stages. Red arrowhead represents the timing of *EDA24-like* expression. **(B–E)** Micrographs of wild-type and ABAP1^OE^ ovules. Embryo sacs and embryo sac cells were artificially outlined for better visualization. DIC microscopy **(B)** and DAPI staining **(C)** of wild-type ovules indicate the central cell nucleus (Ccn and white arrowhead) formed by fusion of the two polar nuclei. DIC microscopy **(D)** and DAPI staining **(E)** of ABAP1^OE^ ovules show two unfused polar nuclei (PN and red arrowheads). EC, egg cell; Ccn, central cell nucleus; PN, polar nuclei. Scale bars at **(B–E)** represent 20 μm. **(F)** Percentage of unfused polar nuclei quantified in emasculated flowers at stage 13 (FG6-FG7) from wild-type Col, ABAP1^OE^ and *eda24-like^*KD*^* plants. For WT, 6 ovaries were analyzed, with a minimum of 25 and a maximum of 39 ovules analyzed per ovary. For ABAP1^OE^, 14 ovaries were analyzed, with a minimum of 25 and a maximum of 36 ovules analyzed per ovary. For *eda24-like*^KD^, 8 ovaries were analyzed, with a minimum of 23 and a maximum of 34 ovules analyzed per ovary. A statistical analysis was performed by *t*-test (*p*-value < 0.05). Asterisks (*) indicate significant changes. **(G)** Relative mRNA levels of *ABAP1, Agamous 80*, *CCG, Agamous 61* and *EDA24-like* were determined by qRT-PCR in wild-type and ABAP1^OE^ flower buds. Data were normalized with *UBI14* as reference gene and were compared with wild-type. Each biological replicate was performed with a pool of 15 flower buds. Bars indicate mean ± standard error of biological replicates. A statistical analysis was performed by *t*-test (*p*-value < 0.05). Asterisks (*) indicate significant changes.

To test whether defects on female gametophyte development could also be contributing to the reduced fertility of ABAP1^OE^ plants, embryo sac differentiation was analyzed. Flower buds of ABAP1^OE^ plants were emasculated and the embryo sac structure was analyzed at stage 13 of flower development (Smyth et al., 1990). At this stage, seven nuclei were visible in wild-type mature embryo sac corresponding to two synergids and the egg cell in the micropyla, the three antipodal cells in the chalaza and the 2*n* fused nuclei of the central cell in the median portion of the embryo sac (Figures 5A–E). Surprisingly, ovules from ABAP1^OE^ flowers had eight instead of seven nuclei in the embryo sac due to unfused polar nuclei (Figures 5D,E). The polar nuclei were still present in 44% of ABAP1^OE^ ovules in FG6 (seven celled) and FG7 (four celled) stages (Christensen et al., 1997), when the diploid central cell should have been formed (Figure 5F).

The data suggested that ABAP1 is not required to regulate the mitotic divisions of the female gametogenesis but seems to be important in events regulating nuclear fusion (Figures 5D,E). A possible role of ABAP1 in the female gamete differentiation is further supported by the presence of ABAP1 protein in the embryo sac observed by immunolocalization experiments (Figures 2J,K), as well as its up-regulation in this structure observed in ABAP1^OE^ plants (Supplementary Figures 2E–H).

### ABAP1^OE^ Down-Regulates Expression of Several Genes in Flower Bud

To get insights on the molecular mechanisms underlying the defects of male and female gametogenesis in ABAP1^OE^ plants, transcripts of ABAP1^OE^ and wild-type flower buds were compared in a microarray experiment. The studies were performed with flower buds of stage 13, since polar nuclei are already fused at this developmental stage (Smyth et al., 1990).

247 genes were differentially expressed in ABAP1^OE^ plants, of which 226 were down regulated (Supplementary Table 1 and Supplementary Figure 6A). This is consistent with ABAP1’s role as a transcriptional repressor (Masuda et al., 2008). To validate the microarray data, the expression of some of these genes was analyzed by qRT-PCR (Supplementary Figure 6B) revealing that they were in good agreement with those from the microarray experiment. GO term network was done using Cytoscape and showed an enrichment of GO terms (*p* < 0.05) related to transport, GTPase activity and pollen development/pollen tube growth in the down regulated genes of the array (Supplementary Figure 7A). This result is expected since male gametophyte development, and therefore the downstream events such as cell morphogenesis and pollen tube growth, was impaired in ABAP1^OE^ plants. GO term enrichment in down regulated genes related to molecular function and cellular components showed genes involved in the endomembrane system as well as genes with pectinesterase and enzyme inhibitor activities (Supplementary Figures 7B,C).

Several *Arabidopsis* mutants with polar nuclear fusion defects were already characterized (Pagnussat et al., 2005; Portereiko et al., 2006b; Maruyama et al., 2016). One of these mutants is a pectinesterase inhibitor and was named *eda24* (*embryo sac development arrest 24*) (Pagnussat et al., 2005). Although *EDA24* gene expression was not altered in our array, its closest *Arabidopsis* homolog was down regulated in ABAP1^OE^ flower buds. This gene was still uncharacterized and was named *Embryo Sac Development Arrest 24-like* (*EDA24-like*, AT1G23350). Thus, a possible participation of EDA24-like in the polar nuclei phenotype observed in ABAP1^OE^ ovules was addressed. To evaluate the defect in embryo sac maturation of ABAP1^OE^ plants, expression of ovule development marker genes and *EDA24-like* were investigated in ovaries through qRT-PCR. The mRNA levels of *Agamous-like80* (*AGL80*), a transcription factor involved in central cell maturation (Portereiko et al., 2006a), and of the *Central Cell Guidance* (*CCG*), expressed at the final maturation step of embryo sac and that guides the pollen tube (Chen et al., 2007), were slightly but significantly reduced in ABAP1^OE^ ovary (Figures 5A,G). A more drastic reduction in the expression level of *Agamous-like61/DIANA*, expressed exclusively at the central cell and early endosperm (Steffen et al., 2007; Bemer et al., 2008), and of *EDA24-like* were observed in ABAP1^OE^ (Figures 5A,G), confirming the reduction of *EDA24-like* expression in ABAP1^OE^ with unfused polar nuclei.

To assess the role of EDA24-like in the female gametophyte development, a SALK T-DNA insertion line with a 66% reduction in *EDA24-like* expression levels was analyzed and hereafter referred as *eda24-like^*KD*^* (Supplementary Figure 4C). Remarkably, around 40% of the ovules in *eda24-like^*KD*^* showed defects in polar nuclei fusion at FG6-FG7 stages, when the diploid central cell should have been formed (Figure 5F). This result is similar to the one observed in ABAP1^OE^ ovules, strongly supporting that down regulation of *EDA24-like* might be contributing to the deficiency in embryo sac development observed in ABAP1^OE^ plants.

### ABAP1 Interacts With ADAP and Regulates EDA24-Like Levels

**FIGURE 6:**
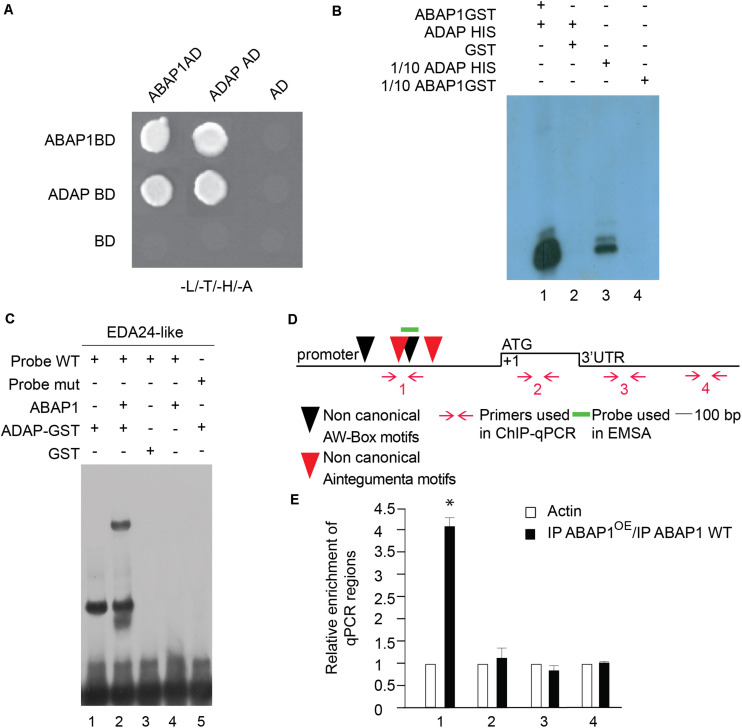
Characterization of ABAP1 and ADAP interaction, and their role in transcription. **(A)** Yeast two hybrid interaction assays between ABAP1 and ADAP. Negative controls used AD and BD empty vectors. The panel shows conjugated yeasts growing in SD medium lacking leucine, tryptophan, histidine and adenine, for selection of strong protein interactions. **(B)** GST-pull down assay between ABAP1–GST and ADAP-HIS produced in *E. coli*. Western blot analysis used anti-HIS antibody. Lane 1, ADAP-HIS with ABAP1-GST; lane 2, ADAP-HIS with GST alone; lanes 3 and 4, 1/10 inputs of ABAP1-GST and ADAP-HIS, respectively. **(C)** EMSA of ABAP1, ADAP and the complex ABAP1-ADAP with wild-type (WT) and mutated (mut) radiolabeled probes of *EDA24-like* promoter region harboring ADAP recognition box. Lane 1, ADAP binds to *EDA24-like* WT probe; lane 2, addition of ABAP1 causes a supershift in the ADAP binding to *EDA24-like* WT probe; GST alone (lane 3) or ABAP1 alone (lane 4) do not bind to *EDA24-like* WT probe; lane 5, ADAP does not bind to *EDA24-like* mutated probe. **(D,E)** Chromatin immunoprecipitation of wild-type Col (WT) and ABAP1^OE^ flower buds with anti-ABAP1 antibody. **(D)** Schematic representation of the amplified regions of *EDA24-like* gene. ANT non-canonical motifs (located at positions –682 to –696 bp and –892 to –905 bp) are represented as red arrowheads. AW-box non-canonical motifs (located at positions –828 to –842 bp and –1,151 to –1,166 bp) are represented as black arrowheads. Red arrows and numbers indicate primers used for ChIP-qPCR assays, and short green line indicates DNA probe used for EMSA. The translational start site (ATG) is shown at position +1. **(E)** Chromatin immunoprecipitated with anti-ABAP1 antibody was analyzed by qPCR using gene-specific primer sets, numbered as indicated in **(D)**. The graph shows the fold enrichment of qPCR amplified regions at the promoter (*1*), coding region (*2*) and 3′UTR (*3,4*) of *EDA24-like*. The fold enrichment of each amplified region was calculated as a ratio between IP ABAP1^OE^ and IP WT of values normalized with values of the *ACTIN 2* promoter. Error bars represent the SD of three biological replicates. Asterisk (^∗^) indicates significant difference from the enrichment according to Student’s *t*-test (*p*-value < 0.05). IP, immunoprecipitated with anti-ABAP1 antibody.

ABAP1’s role as transcriptional repressor involves the association with a transcription factor (Masuda et al., 2008). To get insights into a possible partner of ABAP1 in the control of *EDA24-like* expression, the promoter region of *EDA24-like* was searched for transcription factors binding motifs. Two sequences of the non-canonical recognition sites of AINTEGUMENTA (ANT) [gCAC(A/G)N(A/T)TcCC(a/g)ANG(c/t)] (Nole-Wilson and Krizek, 2000) were found at positions −682 to −696 bp, −892 to −905 bp; and two non-canonical recognition sites of WRINKLED 1 (WRK1), the AW-box (CNTNG[(N)7]CG) (Maeo et al., 2009) were found at positions −828 to −842 and −1,151 to −1,166, suggesting a possible regulation of *EDA24-like* expression by APETALA2 (AP2)-type transcription factors (Figure 6D).

Remarkably, an AP2-type transcription factor that interacts with ABAP1 was identified in a two-hybrid screen using ABAP1 as bait (Figure 6A). This gene was previously identified as ADAP [ARIA-interacting double AP2-domain protein/WRINKLED3 (WRI3)], involved in seedling growth, ABA responses and in the regulation of the fatty acid biosynthetic pathway (Lee et al., 2009; To et al., 2012). For simplification, this gene will be referred to as *ADAP* from now on in this document. Interestingly, ADAP is an interactor of ARIA, the only ABAP1 homolog in *Arabidopsis*, and is also expressed in synergids, egg cell and central cell, according to data publicly available from microarray experiments (Wuest et al., 2010), supporting that ADAP and ABAP1 might co-localize and interact during embryo sac differentiation. We thus investigated whether ADAP also plays a role in gametogenesis, especially regulating *EDA24-like* expression. The interaction between ABAP1 and ADAP was further confirmed by a GST-pull down assay with ADAP–HIS and ABAP1-GST (Figure 6B) and in a semi *in vivo* HIS-pull down experiment, where His-fused ADAP were added to *Arabidopsis* protein extract and used in nickel column purification (Supplementary Figure 4A). In the GST pull down, ADAP associated with ABAP1–GST (Figure 6B, lane 1) but not to GST alone (Figure 6B, lane 2). The ability of ADAP-ABAP1 complex to recognize *EDA24-like* promoter and regulate its expression was first investigated in an EMSA performed with a probe containing the AW-box of the *EDA24-like* promoter region (Supplementary Information). ADAP alone and ADAP–ABAP1 associated with the wild-type probes (Figure 6C, lanes 1 and 2) but ADAP alone did not associate with the mutated probe (Figure 6C, lane 5). ABAP1 alone or GST did not associate with ADAP consensus motif probe (Figure 6C, lanes 3 and 4). The association of ABAP1-ADAP complex with *EDA24-like* promoter was also confirmed *in vivo* in ChIP- PCR experiments with anti-ABAP1 antibody (Supplementary Figure 5C2 and Figure 6D), using chromatin extracts from *Arabidopsis* ABAP1^OE^ and wild-type flower buds. ABAP1-ADAP interaction was detected in the *EDA24-like* promoter of wild-type plants (Supplementary Figure 5C2, lane 3). Next, four different primer sets were designed to amplify regions along the *EDA24-like* promoter, coding sequence and 3’UTR regions. Primer set number one was specifically designed to amplify a region that harbors both the AINTEGUMENTA and the AW-box motifs (Figure 6D) in the promoter region of *EDA24-like* gene. The results showed that ABAP1 associated with the region of the *EDA24-like* promoter that contains the AW-box in wild-type flower buds and that this association was enhanced in ABAP1^OE^ (Figure 6E).

The data suggests that ABAP1 associates with ADAP in flowers, and the ABAP1-ADAP complex negatively regulates *EDA24-like* expression, controlling the polar nuclei fusion during maturation of the female gametophyte. Similar effects on central cells’ formation were observed in ABAP1^OE^ and *eda24-like^*KD*^* plants, supporting this model of action of ABAP1 during female gametogenesis.

## Discussion

During higher plants’ life cycle, the diploid sporophyte body and the haploid gametophytes establish unique strategies to modulate their development. It implies that they might have evolved particular mechanisms to connect the cell cycle progression with developmental and environmental signals. ABAP1 is a plant specific protein that has been previously implicated in the control of cell proliferation homeostasis during vegetative leaf development (Masuda et al., 2008). Here, we characterized plants with up-regulated levels of ABAP1, and studied ABAP1’s association with different transcription factors, repressing the expression of their target genes during plant reproduction. We propose novel regulatory mechanisms in which ABAP1 is involved, by fine-tuning (i) formative cell division in male gametophyte and (ii) cell differentiation (or cell fusion) in female gametophyte, two processes that are essential for a successful plant reproduction in *Arabidopsis*.

### ABAP1 Participates in the Regulation of the Formative Cell Division During Differentiation of Male Gametophyte

During the formation of the male gametophyte in flowering plants, the haploid microspore generated after meiosis undergoes two rounds of mitotic divisions in order to form the mature male gametophyte. The first asymmetric mitotic division (Pollen Mitosis I, PMI) results in a large vegetative cell and a small generative cell which undergoes a second symmetric cell division yielding the two sperm cells (Twell, 2011).

The successful progression of pollen development requires the function of genes specifically active in the haploid gametophyte, as well as of genes involved in both gametophyte and sporophyte development (Brownfield et al., 2009). There are already a number of genes described as affecting various aspects of pollen development in *Arabidopsis*. Out of those mutants, some male gametophyte mutations affecting PMI and PMII have been characterized, such as *sidecar pollen*, *duo1* (*duo pollen1*), *duo2* (*duo pollen2*), *gem1* (*gemini pollen1*), and *gem2* (*gemini pollen2*) [reviewed in Liu and Qu (2008)].

Some cell cycle regulators of diploid sporophyte have also been shown to control male gametogenesis. The smaller generative cell formed during PMI asymmetric division is further engulfed by the large vegetative cell, a process that requires the presence of the plant retinoblastoma homolog RBR1. Pollen with multiple vegetative cells are formed in *rbr1* mutants (Chen et al., 2009). The CDKA;1 activity is also of key importance for PMII. The *cdka;1* mutant pollen develops a vegetative cell similar to the wild-type, but with only one generative/sperm cell-like (Nowack et al., 2006). A similar phenotype was also observed in mutants of the F-BOX-LIKE 17 (FBL17) gene, that acts together with SKP-CULLIN-F-BOX (SCF) complex mediating the degradation of KRP6 and KRP7 (Gusti et al., 2009). However, a detailed molecular genetic framework of cell-cycle control over male gametophyte development is still missing in plants.

Besides regulating the balance of proliferative mitotic divisions during vegetative growth, our data suggest a novel role of ABAP1 in formative cell division during male gametophyte development. Here we have shown that ABAP1, having TCP16 as its partner, negatively regulates *CDT1b* transcription, possibly affecting pre-RC assembly, consequently DNA replication and cell division. We showed that PMI was affected in *cdt1b*^*KD*^ mutants (Figure 3A, bottom line), similarly as in ABAP1^OE^ plants. CDT1a and CDT1b were previously shown to have partially redundant functions during male gametophyte development in *Arabidopsis*, however, the function of CDT1b alone was not investigated (Domenichini et al., 2012). Moreover, only CDT1a had a role in the female gametophyte development, since *cdt1a* mutants showed defects occurring in the mitosis during embryo sac maturation (Domenichini et al., 2012). Remarkably, we found that ABAP1 associated only with the *CDT1b* but not with the *CDT1a* promoter in *Arabidopsis* flower buds (Supplementary Figure 5), indicating that ABAP1 regulates female gametophyte development in a mechanism that does not involve repression of *CDT1a* expression, as discussed below.

The data indicates that ABAP1 acts during male gametogenesis in a similar way to those previously described in leaves, as it also involves repression of *CDT1b* expression. Nevertheless, in this different developmental context, the TCP partner, a member of class I TCP transcription factors, seems to be negatively regulating cell cycle progression of the male gametophyte (Li et al., 2012; Aguilar-Martínez and Sinha, 2013). Acting as a negative regulator of mitotic cell cycle progression, ABAP1 possibly integrates the timing of the male gametophyte differentiation with developmental cues.

### ABAP1 Has a Role in the Female Gametogenesis Differentiation

In most angiosperm species, including *Arabidopsis*, after meiosis the female gametophyte undergoes three rounds of mitosis followed by cellularizations to produce a seven-celled embryo sac consisting of one egg cell, two synergids, three antipodal cells, and one diploid central cell. In *Arabidopsis* and other species, the polar nuclei fuse before pollination to form the secondary nucleus of the central cell. Although increasing knowledge about female gametophyte development has been revealed, little is known about polar nuclei fusion. Here, we showed that plants with high levels of ABAP1 yielded mature ovules with unfused polar nuclei due to *EDA24-like* repression mediated by ABAP1-ADAP binding to its promoter sequence.

ADAP is a member of the AP2/ERF family of transcription factors and belongs to basal AINTEGUMENTA (ANT) being related to WRINKLED, rather than to ANT, PLETHORA, AIL, and BABY BOOM (Horstman et al., 2014). It binds to ARIA, the only ABAP1 homolog in *Arabidopsis* and a ABF2-interacting partner that controls ABA response possibly by the same pathway of ABF2 (Lee et al., 2009). Interestingly, ADAP overexpressing lines had fewer and shorter siliques with fewer ovules, a phenotype similar to what was observed in ABAP1^OE^ plants. Analogous to what was proposed for ABAP1-TCP24 interaction, in which the heterodimer downregulates TCP24 target genes (Masuda et al., 2008), our data points to a similar molecular mechanism with ABAP1-ADAP heterodimer repressing ADAP target genes, in this case *EDA24-like*. Downregulation of *EDA24-like* expression observed in both ABAP1^OE^ plants and *eda24-like^*KD*^* knockdown mutants led to the failure of the polar nuclei fusion to form the central cell from FG5 to FG7. This phenotype is similar to the one reported for *eda24* mutants, in which female gametophyte is arrested at two polar nuclei phase (Pagnussat et al., 2005).

Several mutants of cell cycle genes have been described to impair female gametogenesis, such as members of APC/C (Capron et al., 2003; Kwee and Sundaresan, 2003; Pérez-Pérez et al., 2008; Wang et al., 2012, 2013), cyclin/CDK pathway (Nowack et al., 2006; Takatsuka et al., 2015) and G1/S transition genes like RBR1 and pre-replication complex genes (Holding and Springer, 2002; Ebel et al., 2004; Domenichini et al., 2012). The phenotypes observed in these mutants are usually related to cell division or polarity defects rather than to failure in polar nuclei fusion or DNA replication.

Failure in polar nuclei fusion was already described in several other mutants whose molecular function is involved in fatty acid metabolism, RNA metabolism, endomembrane system, mitochondrial metabolism and cytokinin signaling pathway (for review, see Maruyama et al., 2016; Yuan et al., 2016; Liu et al., 2017). Other mutants were also described (*eda24* to *eda41*) but their molecular function is still uncharacterized (Pagnussat et al., 2005). EDA24 is a member of the plant invertase/pectin methylesterase inhibitor superfamily whose function in polar nuclei fusion still remains to be elucidated. Since EDA24 and EDA24-like are both invertase/pectin methylesterase inhibitors, it is possible that pectin metabolism is important during nuclei fusion.

Interestingly, none of the mutant genes defective in polar nuclei fusion described so far were involved in DNA replication control. In *A. thaliana*, it was suggested that the fusion of the male and female chromatin requires both nuclei to be at the same stage of the cell cycle, which occurs at G2/M phase. Since central cell chromatin seems to be at the G2/M transition, it is tempting to speculate whether the fusion of the two polar nuclei also requires the concurrence of ploidy levels, and a passage through S phase (Friedman, 1999; Faure et al., 2002; Berger et al., 2008; Ronceret et al., 2008). ABAP1 has been shown to play a role in S-phase progression in cell proliferation during leaf development (Masuda et al., 2008). Here, we also report a similar mechanism occurring in ABAP1^OE^ plants, in which ABAP1 represses *CDT1b* transcription, possibly arresting the unicellular pollen at G1/S. Unfortunately, information about ploidy level of female gametophyte cells is still missing. If the polar nuclei have to be at G2/M to form the central cell, then, nuclei arrested at G1/S could partially explain the defect observed in ABAP1^OE^.

### ABAP1 Is Involved in Mechanisms That Regulate Differentiation of Both Male and Female Gametophytes

The underlying molecular and biochemical mechanisms coordinating the complete fertilization process in flowering seed plants (angiosperms) are recently being studied and revealed. During self-fertilization in compatible conditions, an intriguing issue is how the differentiation of the two female gametes (egg and central cells) and the two male sperm cells are coordinated, since these two events must be synchronized in different plant organs to allow a timely double fertilization.

Our data revealed that ABAP1 is involved in mechanisms that regulate differentiation of both male and female gametophytes, as the up-regulation of ABAP1 levels arrested male gametogenesis at the first gametophyte cell division and prevented fusion of the two polar nuclei in the central cell. An interesting question to be addressed is whether ABAP1 could participate in mechanisms that coordinate the timing of the gametophyte differentiation. The transcriptional regulators RBR1/E2Fa play a dual role in male and female gametogenesis by controlling the cell-cycle progression in both gametophytes. RBR1/E2Fa directly regulates the F-box protein FBL17, in a negative regulatory cascade where FBL17 inhibits CDKA;1 (Desvoyes et al., 2014). Accordingly, concomitant loss of CDKA;1 and FBL17 resulted in female and male gametophytes with defects in the mitosis post-meiosis (Zhao et al., 2012).

As male gametophyte differentiates in vegetative and sperm cells and the seven-celled female gametophyte is formed, the two sperm cells have to be guided toward the two female gametes. Great advances have recently been made in the understanding of the regulation of pollen tube guidance toward the two female gametes. The synergid cells and the egg cell of the seven-celled female gametophyte communicate with each other to establish and maintain their identity (reviewed in Skinner and Sundaresan, 2018). These four cell types have also been shown to generate and secrete signaling molecules, such as multiple peptides and especially small Cys-rich proteins, that are sensed by receptor-like kinases in a mechanism involved in guiding the male gametophyte (reviewed in Higashiyama and Yang, 2017). The central cell was also implicated in playing a role in pollen tube guidance since mutants for a gene expressed in these cells, named the *CCG*, are defective in pollen tube attraction (Chen et al., 2007). As ABAP1^OE^ female gametophytes did not form a one-nucleus central cell, ABAP1 might be involved in regulating this step of female gamete differentiation. Defects in pollen tube attraction and reception as well as zygote activation could also be occurring in ABAP1^OE^ plants but were not addressed on this work.

After the sperm cell discharge, gamete fusion of the male and female chromatins (karyogamy) might require a cell cycle synchronization. In *Arabidopsis*, it has been suggested that the karyogamy occurs with nuclei at G2/M phase. At the end of pollen maturation, the two male sperm cells seem to reinitiate the S phase, progressing into the cell cycle until being arrested at the G2/M transition (Durbarry et al., 2005). For the female gametes, it has been proposed that the central cell arrests at the G2/M transition, whereas the egg cell arrests at the G1/S transition and has to progress into the cell cycle prior to gamete fusion, as the first zygote mitosis happens 16 h after fertilization and requires transcription of the thymidylate kinase at the G1/S transition (Faure et al., 2002; Berger et al., 2008; Ronceret et al., 2008). As discussed above, it is tempting to speculate that ABAP1, as a DNA replication repressor (Masuda et al., 2008), could be participating in a mechanism that controls the timing of the last steps of male and female gametes maturation, by regulating S phase progression, as coordination of cell cycle synchronization is necessary for karyogamy and polar nuclei fusion.

The different effects of ABAP1 de-regulation on gametophyte development are intriguing. We have shown that specific ABAP1 transcription factor partners might account, at least in part, for the different phenotypic outcomes of the male and female gametophytes. Although our data show effects of ABAP1 imbalance in the gametogenesis, we cannot rule out possible effects of ABAP1 in sporophytic tissues that might indirectly affect gametophyte development. Interestingly, down regulation of ABAP1 did not affect gametogenesis. This could result from the accumulation of maternally inherited ABAP1 protein. Otherwise, since ABAP1 is a repressor of the basic machinery that licenses DNA to replicate, it works by regulating the rate at which the cell cycle progresses into G1/S phase, being possibly a sensor of internal and external conditions. Thus, another possibility is that ABAP1 is only necessary to prevent and/or slow down gametophyte development in specific environmental conditions.

**FIGURE 7:**
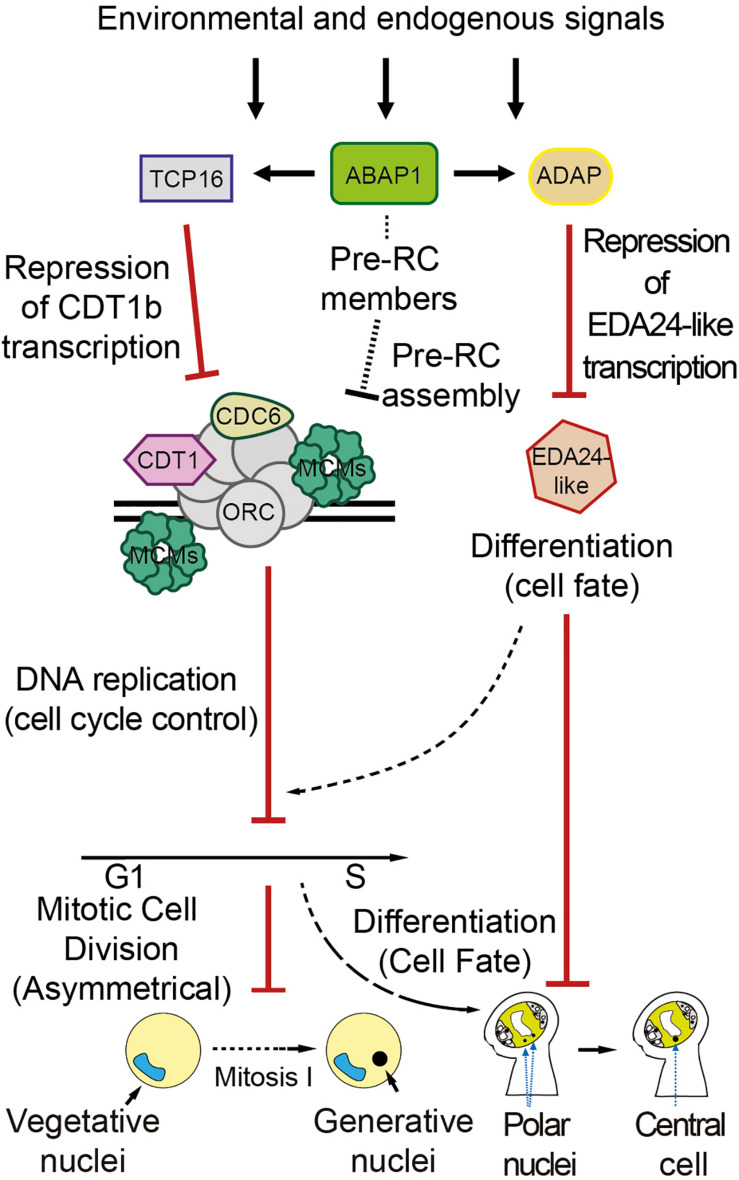
Proposed model for ABAP1 role in the differentiation of male and female gametophytes. ABAP1, associated with transcription factors partners, might sense environmental and endogenous signals, triggering changes in gene expression patterns that will regulate male and female gametophytes differentiation. The molecular mechanism operating in the male gametophyte is similar to those previously described in leaves. In the first asymmetric cell division after meiosis, ABAP1 associates with TCP16, represses *CDT1b* transcription, controlling CDT1b homoeostasis. Lower levels of CDT1b might affect pre-RC functioning, limiting DNA replication. A direct association of ABAP1 with pre-RC members is still not determined. However, as described in leaves, it could also affect pre-RC assembly and DNA replication in the male gametophyte. In this way ABAP1 would regulate the timing of cell cycle progression at G1 to S. The mechanism of action in the female gametophyte involves association with another partner, ADAP, during embryo sac maturation. ABAP1-ADAP also represses gene transcription of *EDA24-like* that is necessary for polar nuclei fusion. Thus, ABAP1 might regulate the timing of polar nuclei fusion to form the central cell, that is crucial in pollen tube guidance. The involvement of DNA replication in the mechanism that regulates female differentiation is still not determined.

In this work, we have determined the molecular and biochemical mechanisms by which ABAP1 is involved in the regulation of male and female gametogenesis, which comprise binding to different transcription factors and controlling their gene expression targets (Figure 7). It remains to be determined whether ABAP1 might also participate in a mechanism that regulates the timing of male and female gametophytes differentiation in *Arabidopsis*, integrating it with environmental and internal signaling, to ensure the reliable and on-time fusion of the two pairs of gametes. The double-fertilization has countless biological and agricultural implications and mechanisms that guarantee a successful double fertilization might provide selective advantage for plants. Also, effective plant fertilization is essential for an efficient seed production, thus molecular understanding of the mechanisms involved in controlling double fertilization could provide tools to improve plant yield.

## Data Availability Statement

Sequence data from this article can be found at the Arabidopsis Genome Initiative database with the following accession numbers: ABAP1 (At5g13060), Actin 2 (At3g18780), UBQ14 (AT4G02890), CDT1a (At2g31270), CDT1b (At3g54710), TCP16 (AT3G45150), EDA24-like (At1g23350), and ADAP/WRI3 (At1g16060). Microarray data was deposited in Gene Expression Omnibus (nos. GEO: GSE164480 and GSM5011985).

## Author Contributions

LC and HM performed the molecular and cellular studies. HB and JA-E performed immunolocalization studies. MA-F carried out the microarray experiments. HM and FB performed the *in silico* analyses. LC carried out the biochemical assays. LC, HM, and AH designed the work and analyzed the data. PF and KD participated in the analyses and discussion of results. LM, HM, and AH wrote the manuscript. All authors read and approved the final manuscript.

## Conflict of Interest

The authors declare that the research was conducted in the absence of any commercial or financial relationships that could be construed as a potential conflict of interest.

## Supplementary Material

Supplementary Figure 1 ABAP1 localization during male gametogenesis in WT plants.

Supplementary Figure 2 ABAP1 localization in pollen and ovary of WT and ABAP1^OE^ plants.

Supplementary Figure 3 Anther and male gametophyte development in ABAP1^OE^ flowers.

Supplementary Figure 4 Semi *in vivo* interaction between ABAP1, TCP16, and ADAP and schematic representation of the genomic T-DNA insertions and gene expression in *cdt1b*^*KD*^ and *eda24-like^*KD*^* mutant lines.

Supplementary Figure 5 Characterization of the interaction between ABAP1-TCP16 with the promoters of *CDT1a* and *CDT1b*.

Supplementary Figure 6 Differentially expressed genes in ABAP1^OE^ flowers, analyzed by microarray.

Supplementary Figure 7 Gene Ontology (GO) term network analysis of down regulated genes in ABAP1^OE^ array based on biological processes, molecular function and cellular component.

Supplementary Table 1 List of genes differentially regulated in flower buds of stage 13 of ABAP1^OE^ plants, compared to wild-type control (Col-0).

Supplementary Information Description of detailed experimental procedures.
