# Supplementary material for: ABAP1 Plays a Role in the Differentiation of Male and Female Gametes in Arabidopsis thaliana
Source: Front Plant Sci. 2021 Feb 10;12:642758. doi: 10.3389/fpls.2021.642758 (PMC7903899; doi:10.3389/fpls.2021.642758)
Supplement: Supplementary Figure 1 — ABAP1 localization during male gametogenesis in WT plants. [file Data_Sheet_1.pdf]

SUPPLEMENTARY MATERIAL

1- SUPPLEMENTARY DATA

Constructs

The full-length coding regions of *ABAP1*, *TCP16 and ADAP* were PCR amplified with the following primers:

ABAP1-ATTB1: TACAAAAAAGCAGGCTTCACAATGGAGAACCATCCACAAGCGCCA
ABAP1-ATTB2: CAAGAAAGCTGGGTTACTTCAAACCGGAATCCTATATG
TCP16-ATTB1: AAAAAGCAGGCTTCAATGGATTCGAAAAATGG
TCP16- ATTB2 AGAAAGCTGGGTTCAAACTGTGGTTGTGG
ADAP-ATTB1: GGGGACAAGTTTGTACAAAAAAGCAGGCTTCACAATGTTCATCGCCGTCGAAG
ADAP-ATTB2: GGGGACCACTTTGTACAAGAAAGCTGGGTTCAGAAGAACAACACTTTAGCAATCA

Amplified fragments were reamplified with adaptor primers ADAPT-B1 (GGGGACAAGTTTGTACAAAAAAGCAGGCT) and ADAPT-B2 (GGGGACCACTTTGTACAAGAAAGCTGGGT) and cloned into destination vectors described in the paper.

Microscopic Analysis

Plant material was harvested at the indicated developmental stages and fixed with 4% paraformaldehyde in 100 mM sodium phosphate buffer (pH 7.2). For Nomarski and dark field microscopy a Nachet 300 microscope was used. Dark field images were made with fixed material embedded in Historesin (Leica, Wetzlar, Germany) and cutted in semithin sections (3 µm). For light microscopy, the same protocol was used for fixation and slicing the material, and sections were stained with 0.05% toluidine blue and observed under a bright-field Olympus microscope. Alternatively, fixed flowers were dissected under stereosmicroscope and ovules and pollen grains were cleared with lactic acid and chloral hydrate, according to Alexander MP (1987) and stained with 1 ug/mL with DAPI for 5 min and observed in a fluorescence microscope Zeiss LSM550.
For emasculation experiments, flower buds were dissected under stereomicroscope 1-2 days before anthesis, all the stamens were removed and the ovaries were harvested when stigma were receptive. For embryo development experiment, flower buds had all stamens removed under stereomicroscope 1-2 days before anthesis and ovaries were manually fertilized when stigma were receptive. Fertilized siliques at different developmental stages (1- to 24 h after fertilization) were fixed in 4% (v/v) formaldehyde in PBS, dehydrated in ethanol series (10%, 30%, 50%, 70%, 80%, 90%, 95% and 100% twice) for 15 min, transferred to xylene and embedded in paraplast (Sigma). 10 µm sections were made using a rotary microtome (American Optical, USA), and heat fixed to glass slides. Sections were stained with toluidine blue before removal of paraffin with xylene (20 min twice). Bright-field images were analyzed in a Olympus microscope.
In vitro pollen germination was performed according to Ye *et al (2009)*. Pollen was harvested from newly opened flowers and placed onto the pollen germination medium consisting 1 mM $CaCl_2$, 1 mM $Ca(NO_3)_2$, 1 mM $MgSO_4$, 0.01% (w/v) H_3BO_3, and 18% (w/v) sucrose solidified with 0.8% (w/v) agar, pH 7.0. Plates were cultured at 28 °C at high humidity for 8 h.

Promoter GUS experiments

Flowers of 6-weeks-old homozygous ABAP1Pro::GUS plants grown in the soil were used for histochemical localization of GUS activity. GUS activity was detected histochemically with 1 mM 5-bromo-4-chloro-3-indolyl β-D-glucoronide, 10 mM phosphate buffer (pH 7.0), 0.05 mM potassium ferrocyanide, 0.05 mM potassium ferricyanide, 2 mM EDTA, and 0.1% (v/v) Triton X-100 at 37 °C for 4-16 h. The material was cleared with chlorolactophenol (chloral hydrate:phenol:lactic acid, 2:1:1) and analyzed with differential interference contrast (DIC) optics (Axiophot, Zeiss, Göttingen, Germany). Alternatively, plant material was fixed with 4% paraformaldehyde in 100 mM sodium phosphate buffer (pH 7.2), embedded in Historesin (Leica, Wetzlar, Germany) and cut in 3 µm semithin sections in a rotary microtome (American Optical, USA). Sectioned material was observed under bright-field optics (Olympus® CX31RTS5).

Microarray experiment

Microarrays were based on the Arabidopsis Genome Oligo Set version 1.0 (Operon) that consists of a total of 26,090 oligonucleotides corresponding to 22,361 annotated genes. Microarrays were manufactured as previously described (Wellmer et al., 2004) and was kindly donated by Dr. Elliot Meyrowitz (Caltech, USA).
Three sets of WT and ABAP1OE plants were grown at different moments under the same conditions comprising three biological replicates. Floral buds from at least 20 plants of WT or ABAP1OE were collected in liquid nitrogen at stage 13 of flower development (Smyth et al., 1990) for each biological sample. Total RNA was isolated using the RNeasy RNA Isolation kit (Qiagen) according to the manufacturer's instructions. Dye-labeled antisense RNA was generated from these total RNA preparations and hybridized to microarrays as previously described (Wellmer et al., 2006). The dyes used for labeling RNA from the individual samples were switched in the replicate experiments to reduce dye-related artifacts. Microarrays scanning and raw data processing were performed as previously described (Alves-Ferreira et al., 2007). P values were adjusted using Benjamini and Hochberg procedure in the Bioconductor multtest package. Genes were considered as differentially expressed if they showed an absolute FC value of 2 or more between the wild type and ABAP1OE plants and had been assigned an adjusted P value < 0.05. All analyses in Resolver (Rosetta Biosoftware) were done at the so-called sequence level, i.e. data from reporters (probes) representing the same gene were combined. Cytoscape with BiNGO plugin (Maere et al., 2005) was used to predict genes involved in the same biological processes, in the same organelle and with similar molecular function.

Yeast two-hybrid assay

Yeast strain Y190, with the genotype MATa (gal4, gal80, his3, trp1-901, ade2-101, ura3-52, leu 2-3-112, URA3::GALlacZ, LYS2::GAL(UAS)-HIS3) was co-transformed with 5 µg of the constructs by the Polyethylene glycol/LiAc method (Gietz et al, 1992) and plated on synthetic dropout media without either leucine/tryptophan (-leu/-trp) (to test transformation efficiency) or leucine, tryptophan, and histidine (-leu/-trp/-his) (low stringent condition), or leucine, tryptophan, histidine, and adenine (-leu/-trp/-his/-ade) (high stringent condition), and incubated for 3 days at 30ºC.

In vitro protein interaction assay (GST pull down)

ABAP1-GST, AtTPC24-GST, TCP16-HIS and ANT-HIS were expressed in cells of *E. coli* strain BL21 as described by Chekanova et al (2000), with modification in the lysis buffer (25 mM Tris, pH 8.0, 1 mM EDTA, 10% glycerol, 50 mM NaCl, 0.1% Triton X-100, 1 mM phenylmethylsulfonyl fluoride (PMSF), 10 mM leupeptin, and 75 mM aprotinin). GST-pulldown analyses were carried out according to Tarun & Sachs (1996). We used a Gluthatione Sepharose 4B column (Pharmacia Biothech). The ABAP1 antibody was developed by Covance Corp. using the antigenic peptide GAPIVTQLID (amino acids 57 to 66 from ABAP1 protein) and used 1:1000. The other antibodies used in this paper were GST (1:5000; Sigma, St.Louis, MO) and HIS (1:2000; Santa Cruz Biotechnology).

Electrophoretic mobility shift assay (EMSA)

DNA probes were generated by annealing oligonucleotides spanning the regions of interest and by filling in the single-strand overhangs with α-^{32}P-dCTP using the Klenow fragment. Binding reactions were performed using 50 fmol of each oligonucleotide probe. 50 ng of each recombinant protein, 1x binding buffer (20 mM Hepes-KOH, pH 7.8, 100 mM KCI, 1mM EDTA, 1 mM DTT, 0.05% BSA, and 10% glycerol) and 20 ng of salmon sperm DNA per ml of reaction solution as a non-specific DNA competitor. The mixtures were incubated for 30 min at room temperature and loaded on native 5% polyacrylamide (acrylamide-bisacrylamide, 29:l [w/w]) gels. For electrophoretic mobility shift assays (EMSAs) with specific antibodies, anti-IgG fractions were added to preincubated (1-5 min) reaction mixtures, and the mixtures were incubated for another 30 min at room temperature. Electrophoresis was conducted at 4 V/cm for 40 min with 0.5 X TBE (45 mM Tris-borate and 0.5 mM EDTA, pH 8.2) buffer at room temperature. Gels were dried and autoradiographed using intensifying screens.

Chromatin Immunoprecipitaion assay.

For PCR of IP DNA, input gDNA2 or mock-IP gDNA, 2 µl of ten-fold dilution of immunoprecipitated DNA (25-fold in the case of input DNA) were used as template in a 20µl PCR reaction for a total of 30 cycles of amplification with the primer annealing temperature set at 59ºC. Following PCR amplification 7µl of the PCR reaction was separated on 2% metaphor agarose gels and visualized under UV light using ethidium bromide staining. 1 µl of ten-fold dilution of immunoprecipitated DNA of Whole cell extract, Immunoprecipitated WT and

Immunoprecipitated ABAP1^{OE} were used in qRT-PCR. Primer sequences used in IP assay are available in Tables 3 and 4.

Table 1. Primer sequences used in qPCR experiments.

Gene	Primer forward	Primer reverse	Ref
ABAP1	5'TCAGCCTTAAGAAGAGCTTGCA3'	5'ACCATAATTGAGAGCTGAGCTTAG T3'	1
UBQ14	5'TCACTGGAAAGACCATTACTCTTG AA3'	5'AGCTGTTTTCCAGCGAAGATG3'	2
CDT1a	5’AATCCGATCACTCTTGAAGAAG3’	5’GAACCACGATCTCAAGAAAGCA3’	2
CDT1b	5’AAATGTCGACTGCCGAAACAG3’	5’AAGTGAAATGTCATGTGAAGTTGC TT3’	2
TCP24	5'CTCCACCTCTTGACCACCAT3'	5'TTGGCGAGAGATGAAAGGA3’	1
EDA24-like	5′AGTGATGACTCCATCGTTGTTGA3'	5'CTTCACCTCCGTCTGAGAAGACA3'	*
ADAP	5’ATTGCTAAAGTGTTGTTCTTC3’	5'AAGTTTGAATCTTTTTAAC3'	*
TCP16	5’ATGGTAATGCTACCGCCAGT3’	5’CTCCAAATTGGCCTTCTCAG3’	*
Myb	5′GAACATGGTCGCGGAATC3’	5′GCGTGGGGAATGAGAGTG3′	*
HAP	5′ATGATGCTTGAACGTGACCA3’	5’TCCAGTTGGGATTCGGTTTA3′	*
Agamous80	5′AATTTCACTTACCAATTCATCC3’	5′CACCAACATCAGATCCATGAC3′	*
CCG	5′GCGGGTTTGTGCTAAGGTAG3’	5′CCCAAAATCTGCAAACCAGT3′	*
Agamous61	5′TGAATCTGTATTGGATCGCTACG3’	5′CCCTTCTTCTTCTCTTCTTCTACC3′	*
At4g36600	5'GCGCTGAGCTTTAGAACTGAAAA3 '	5'CGCCTTTCATTGAGAATCTGATT3'	*
At5g06760	5'AAGTTGCATGGGCACAATATTG3'	5'GGACTTCGATCTCATCTAGGTTTTG T3'	*
At2g47040	5'CCCTACCGAAAACGACGTTGT3'	5'GCAGCTGAGGTCAATGACTTCA3'	*
At2g03410	5'AGCCATGGAGATACGAGGATGA3'	5'TAAAGGCAAAATTGATGCAAACAA 3'	*

* This paper
1 Masuda et al., 2008
2 Masuda et al., 2004

Table 2. Primers sequences used in EMSA.

Primer	Sequence
AtCDT1b pro WT	5'-CGTGGCAAATATGGGCCCACAGCTATAGAT-3'
AtCDT1b pro mut	5'-CGTGGCAAATAT**AAA**CCCACAGCTATAGAT-3'
EDA24-like pro WT	5'CACTGAAACATTCCACAATCCGAGACGAAC3'
EDA24-like pro mut	5'CACTGAAACATTCCACAAT**AAA**AGACGAAC3'

Table 3. Primer sequences used in Chromatin Immunoprecipitation assay PCRs

Primer	Sequence	Localization
CDT1b F.a	5’-CGTTTTAACCCCAGTCTTCTGTG-3’	-488 to -465 bp
CDT1b R.a	5’-GCCCATATTTGCCACGTCAC-3’	-151 to -131 bp
CDT1a F.b	5’-AAGCAGCATAAACATTGCACGA-3’	-353 to -331 bp
CDT1a R.b	5’-CGTATACCTCCCGCGCCTAT-3’	- 164 to -144 bp
EDA24-like F	5’-AATCCGAGACGAACATGGAG-3’	-836 to -856 bp
EDA24-like R	5’-AGCATTTTGCCGCTGTTTAT-3’	-1013 to -1033 bp

Table 4. Primer sequences used in Chromatin Immunoprecipitation assay q-RT PCRs

Primer	Sequence	Localization
CDT1b RTF1	5’CAGAAGGCGACTCCATTA3’	-2050 to -2032 bp

CDT1b RTR1	5’GGAATGGGATTCTTCACC3’	- 1916 to -1898 bp
CDT1b RTF2	5’GAATCGGTTTGAGTGACG3’	-163 to -145 bp
CDT1b RTR2	5’ACGAGCTCATTGCTGAAA3’	- 8 to +10 bp
CDT1b RTF3	5’GACTGCCGAAACAGTACG3’	+2000 to +2018 bp
CDT1b RTR3	5’AGGTTGGACCAGCTTAGAA3’	+2149 to +2168 bp
CDT1b RTF4	5’TCGTCATCCAAGCTCAGT3’	+2225 to +2244
CDT1b RTR4	5’GGTTTTAGCGAGGAAAGG3’	+2320 to +2338
EDA24-like RTF1	5’'TTGATGGACTTCACCAAGCA3’	-822 to -802 bp
EDA24-like RTR1	5’CCAGGCTCTCAATCACTTCC3’	- 924 to -904 bp
EDA24-like RTF2	5’ACGCGCTATTGAAGTTGGAC3’	+252 to +271 bp
EDA24-like RTR2	5’GAGTTGATGAAGCGGACGAG3’	+ 175 to +191 bp
EDA24-like RTF3	5’GCTCCCTCCTCCAAATAAAGA3’	+771 to +792 bp
EDA24-like RTR3	5’CTGTTCCATGTCCTAAGTGTT3’	+695 to +716 bp
EDA24-like RTF4	5’AGTAAGATTTGGCGTTGGAGT3’	+1262 to +1283
EDA24-like RTR4	5’ACTCCAACGCCAAATCTTACT3’	+990 to +1011
ACT2 Forward	5’CTTGCACCAAGCAGCATGAA3’	+1321 to 1341
ACT2 Reverse	5’CCGATCCAGACACTGTACTTCCTT3’	+1409 to 1433

2- SUPPLEMENTARY FIGURES

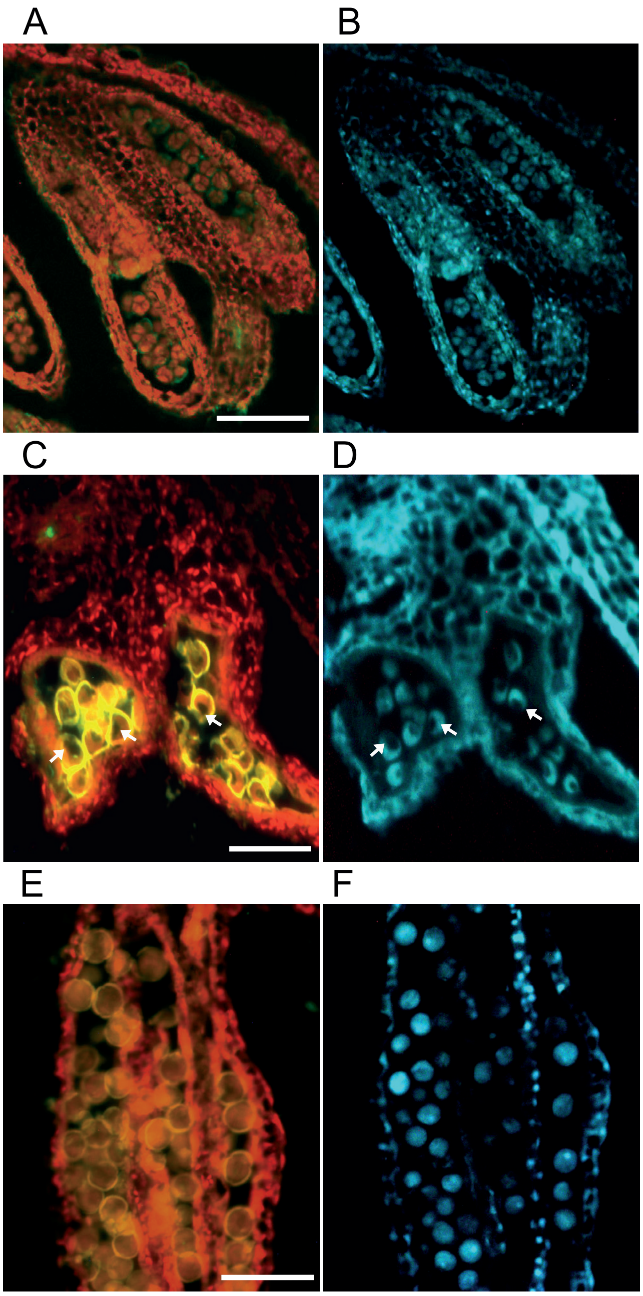

Figure S1 (refers do Figure 2). Pollen development stages in wild-type flowers. **(A-F)** Immuno-localization of ABAP1 protein during pollen differentiation. **(A)** Epifluorescence microscopy of tetrad stages using anti-ABAP1 antibodies conjugated with Alexa Fluor 488, **(B)** DAPI staining reference to A. **(C)** Epifluorescence microscopy of a transversal section of vacuolate stage of microspore, observe the presence of vacuole (arrows) and high fluorescent emission of ABAP1 protein in this stage. **(D)** DAPI staining reference to C. **(E)** Epifluorescence microscopy of a longitudinal section of mature pollen, with reduced fluorescent emission of ABAP1 protein. **(F)** DAPI staining reference to E. Images were taken under the same exposure conditions. The cali-bration bars correspond to 40 µm.

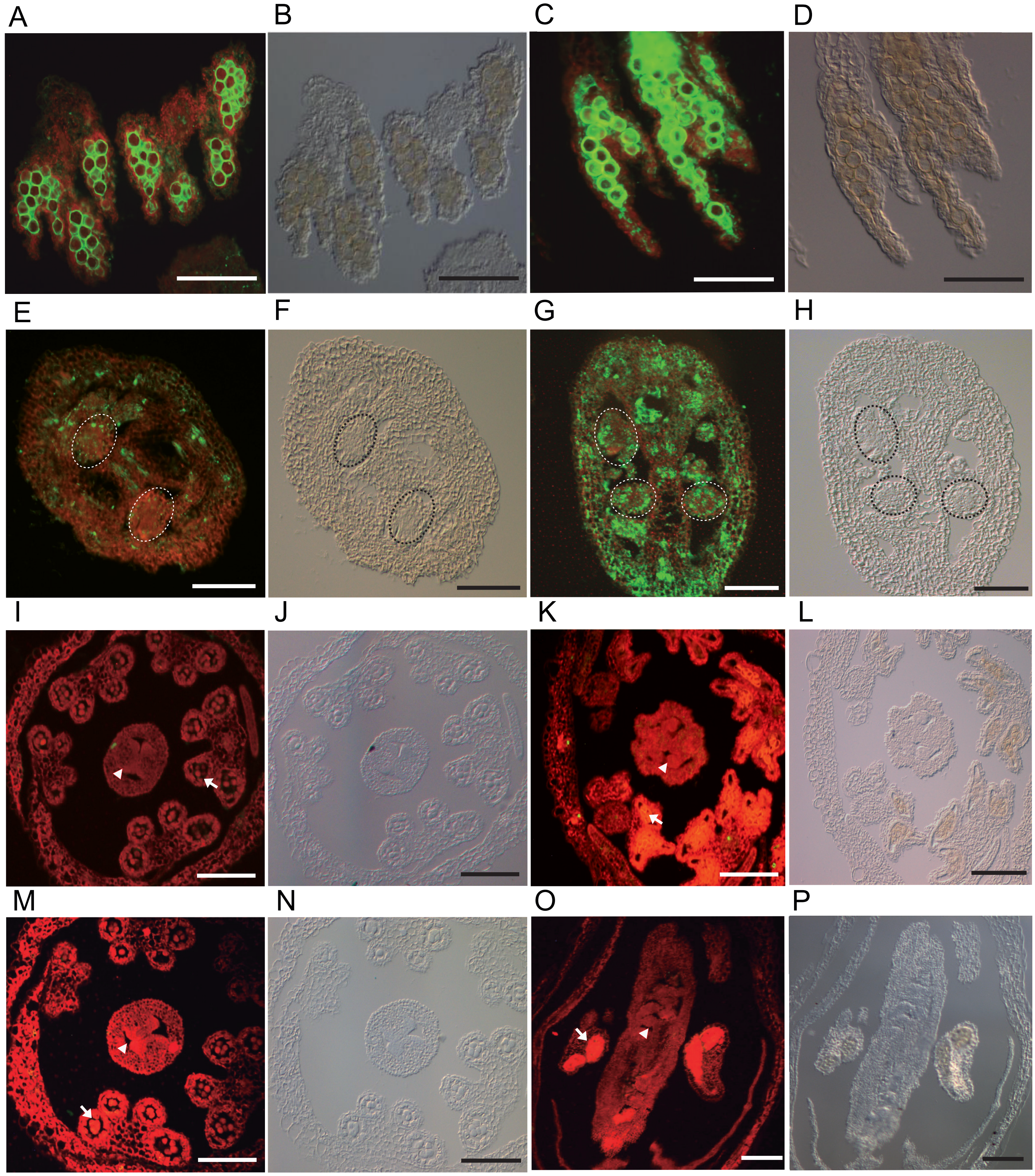

Figure S2 (Refers to Figure 2): ABAP1 protein immunolocalization in Arabidopsis using anti--ABAP1 antibodies conjugated with Alexa Fluor 488. **(A-D)** ABAP1 expression in pollen. **(A)** Epifluorescence microscopy of a longitudinal section of the pollen sac of WT. **(B)** DIC microscopy reference to **(A)**. **(C)** Epifluorescence microscopy of a longitudinal section of pollen sac of ABAP1^{OE}. **(D)** DIC microscopy reference to C. **(E-H)** ABAP1 expression in the ovary. **(E)** Epifluorescence microscopy of WT ovary, showing the presence of ABAP1 in ovules (dashed line). **(F)** DIC microscopy reference to E. **(G)** Epifluorescence microscopy of ABAP1^{OE} ovary. The ABAP1^{OE} ovules (dashed line) have higher levels of ABAP1 protein compared to WT. **(H)** DIC microscopy reference to G. **(I and L)** Epifluorescence microscopy of controls with pre-immune serum (PIS). **(I)** Epifluorescence microscopy of WT control. **(J)** DIC microscopy reference to **(I)**. **(K)** Epifluorescence microscopy of ABAP1^{OE} flower control. **(L)** DIC microscopy reference to **(K)**. **(M and P)** Epifluorescence microscopy of flower controls without anti-ABAP1 and with secondary antibody (Alexa 488). **(M)** Epifluorescence microscopy of WT flower control. **(N)** DIC microscopy reference to M. **(O)** Epifluorescence microscopy of ABAP1^{OE} flower control. **(P)** DIC microscopy reference to O. The pollen (arrow) and ovules (arrowhead) did not emit fluorescence without anti-ABAP1 spe-cific antibody. Images were taken under the same exposure conditions. The calibration bars correspond to 50 µm.

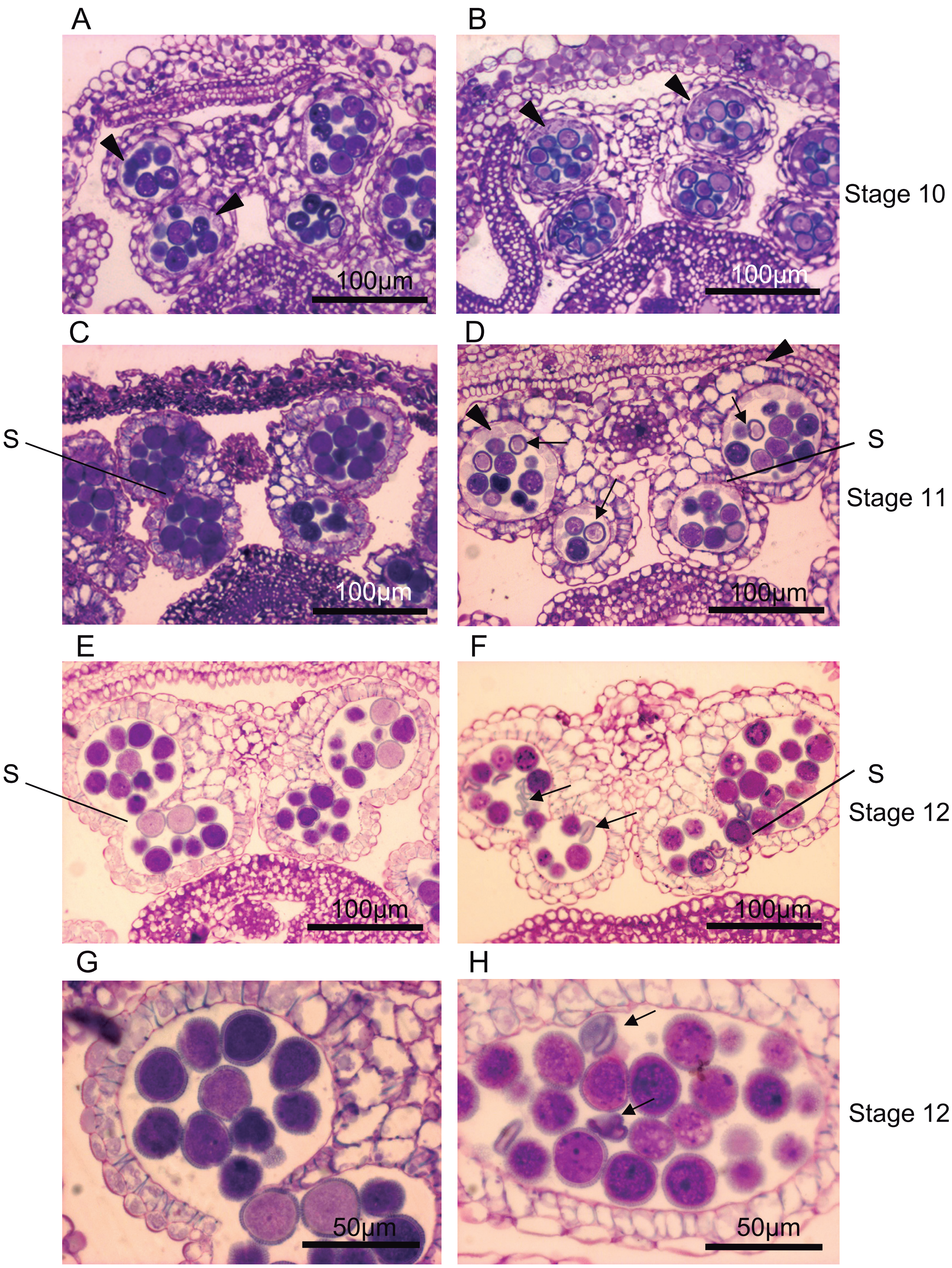

Figure S3 (refers to Figure 3): Anther and male gametophyte development in ABAP1^{OE} flowers. Cross sections of anthers in wild-type **(A, C, E, G)** and ABAP1^{OE} **(B, D, F, H)** flowers. Anther developmental stages are numbered according to Sanders et al. (1999). **(A, B)** Tapetum start degeneration at stage 10 of anther development in both WT and ABAP1^{OE} flowers. **(C, D)** At stage 11, generative cells undergo PM II yielding the two sperm cells and septum starts degeneration. In ABAP1^{OE} anthers, microspore degradation is already observed. **(E, F)** Anthers become bilocular after septum degeneration. Complete degradation of microspores (arrow) are observed in ABAP1^{OE} but not in WT. **(G, H)** Higher magnification of mature microgametophyte observed in WT **(G)**, and completely degraded microspores observed in ABAP1^{OE} **(H)**. Arrowheads indicate tapetum; arrows point to degraded microspores; S, septum. A total 327 pollens from ABAP1^{OE} and 318 from WT plants were counted. 45% and 1.5% of pollen grains were malformed and shriveled in the anthers of the ABAP1^{OE} and WT flowers, respectively.

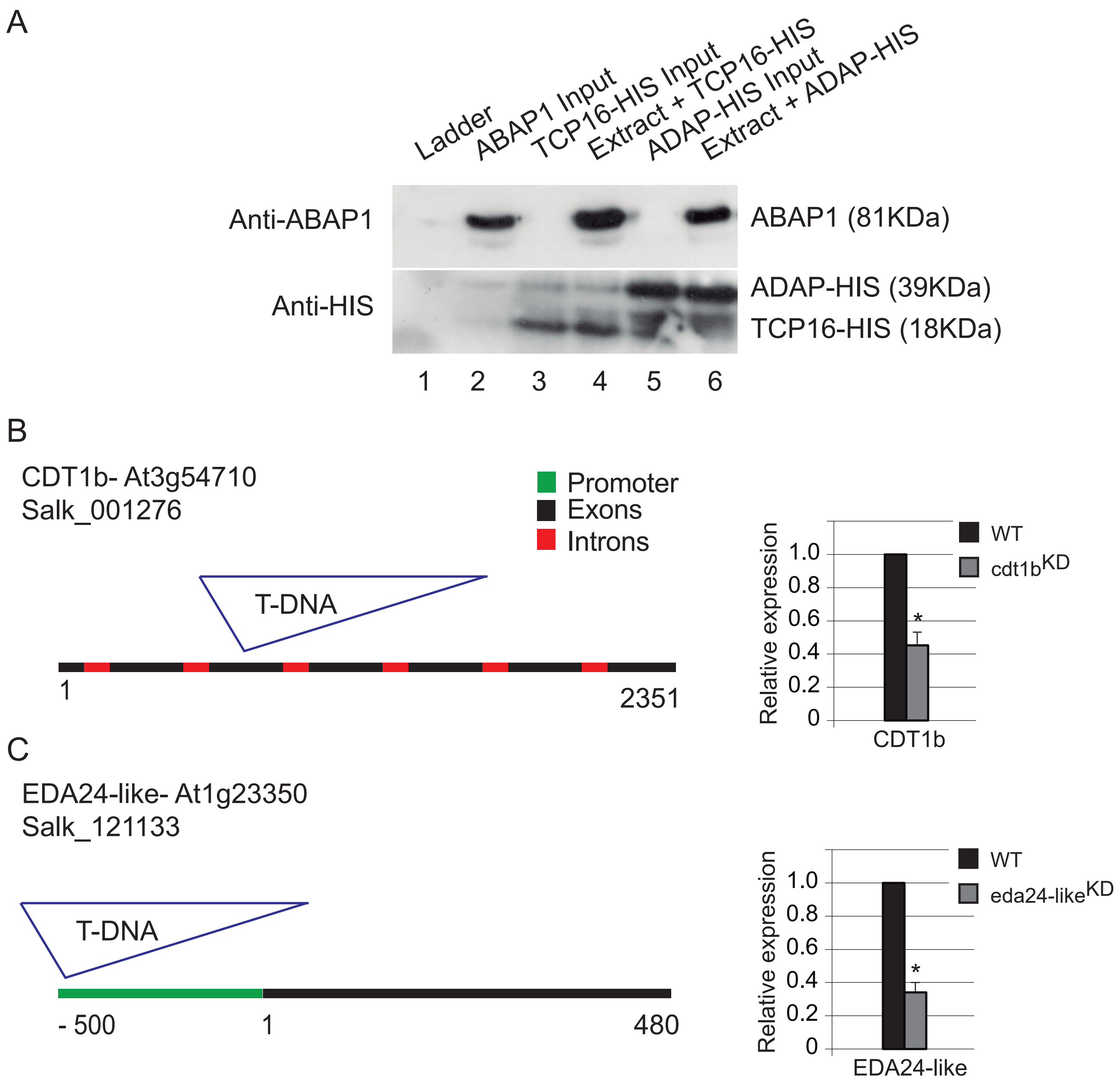

Figure S4 (refers to Figures 4, 5 and 6): **(A)** Semi in vivo HIS-pulldown interaction assay confirming ABAP1 interaction with ADAP and TCP16 in Arabidopsis flowers. TCP16-HIS and ADAP--HIS were mixed with protein extracts of Arabidopsis flowers, purified in a nickel column and tested for the presence of ABAP1 with anti-ABAP1 antibody (upper panel). TCP16 and ADAP recombinant proteins were revealed with HIS-specific antibody (botton panel). The data shows that ABAP1 was purified in the nickel column through interaction with TCP16-HIS and ADAP-HIS fused proteins (lanes 4 and 6, respectively). **(B and C)** Schematic representation of the genomic T-DNA insertions and gene expression in *cdt1b*^{KD} and *eda24-like*^{KD} mutant lines. **(B)** T-DNA insertion in Salk_001276 (*CDT1b*), **(C)** T-DNA insertion in Salk_121133 (*EDA24-like*). The exons are in black, the promoter regions are in green and the predicted introns are in red. The right side B and C shows relative mRNA levels of mutated genes, in wild-type and mutant lines, determined by qRT-PCR. Data were normalized with *UBQ14* as reference gene and were compared with wild--type. Data shown represent mean values obtained from independent amplification reactions (n = 3) and biological replicates (n = 2). Each biological replicate was performed with a pool of 10 plants 20 DAG. Bars indicate mean ± standard error of biological replicates. A statistical analysis was performed by t-test (p-value <0.05). Asterisks (*) indicate significant changes.

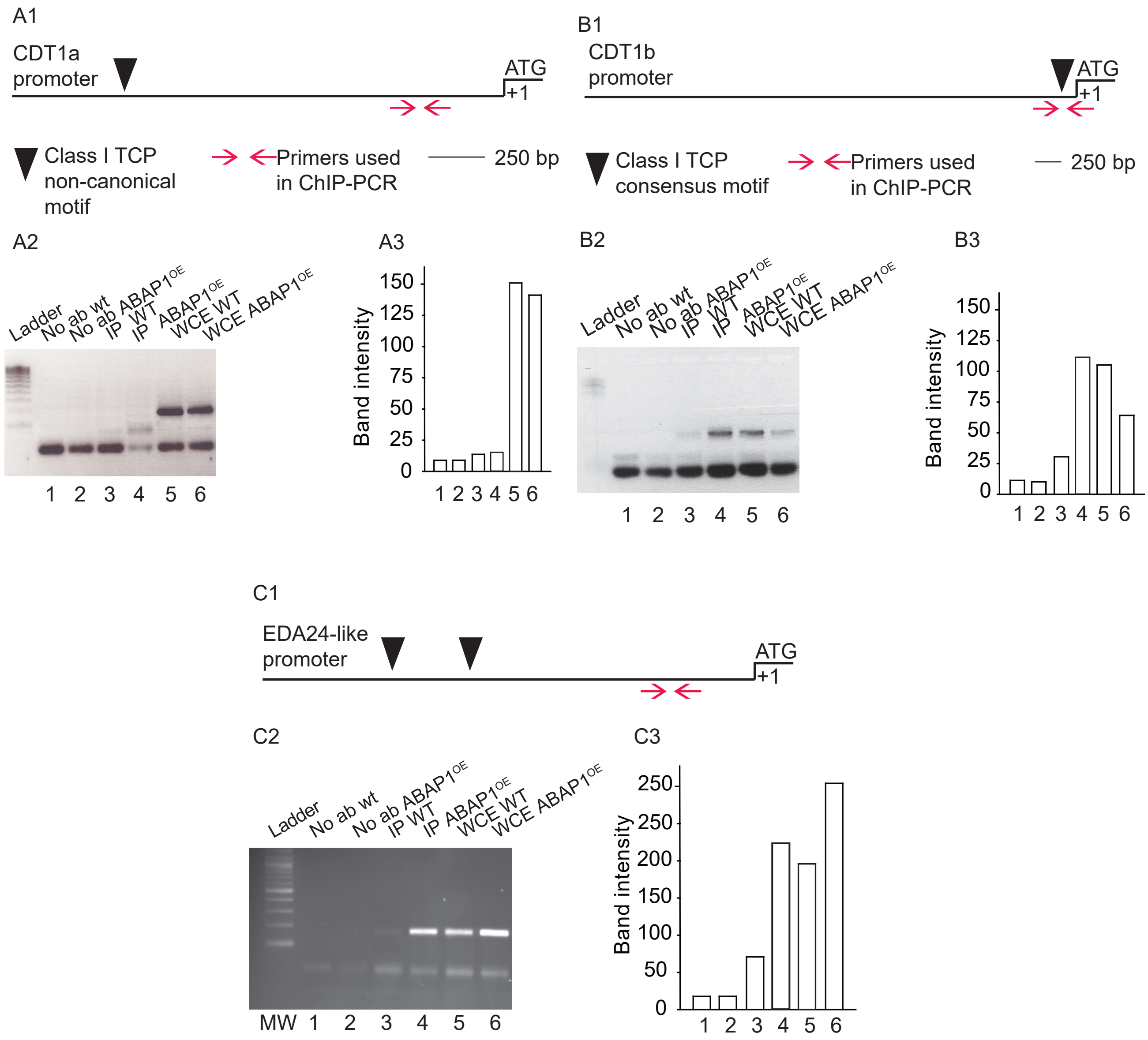

Figure S5 (refers to Figures 4 and 6): Characterization of the interaction between ABAP1-TCP16 with the promoters of *CDT1a* **(A)** and *CDT1b* **(B)** and ABAP1-ADAP with the promoter of *EDA24-like* **(C)**. **(A1)** Schematic representation of the amplified promoter regions of *CDT1a*. Arrows indicate primer sets used in PCR. Class I TCP non-canonical consensus motif, located at position -1335 to -1342 bp, is represented as arrowhead. The translational start site (ATG) is shown at position +1. **(A2)** PCR amplifications of ChIP-gDNA using primer sets for *CDT1a* are shown. PCR band intensities we normalized with lane 2 (no antibody WT) are shown in **(A3)**. **(B1)** Schematic representation of the amplified promoter regions of *CDT1b*. Arrows indicate primer sets used in PCR. TCP I canonical consensus motif, located at position -130 to -136 bp is represented as arrow-head. The translational start site (ATG) is shown at position +1. **(B2)** PCR amplifications of ChIP-gDNA using primer sets for *CDT1b* are shown. PCR band intensities normalized with lane 2 (no antibody WT) are shown in **(B3)**. **(C1)** Schematic representation of the amplified promoter regions of EDA24-like promoter. Arrows indicate primer sets used in PCR. AW-box non-canonical consensus motifs, located at positions -828 to -842 and -1151 to -1166 bp are represented as arrowheads. The translational start site (ATG) is shown at position +1. **(C2)** PCR amplifications of ChIP-gDNA using primer sets for EDA24-like are shown. PCR band intensities normalized with lane 2 (no antibody WT) are shown in **(C3)**.No ab: No antibody; IP: immunoprecipitated with anti-ABAP1 antibody; WCE: whole cell extract.

A

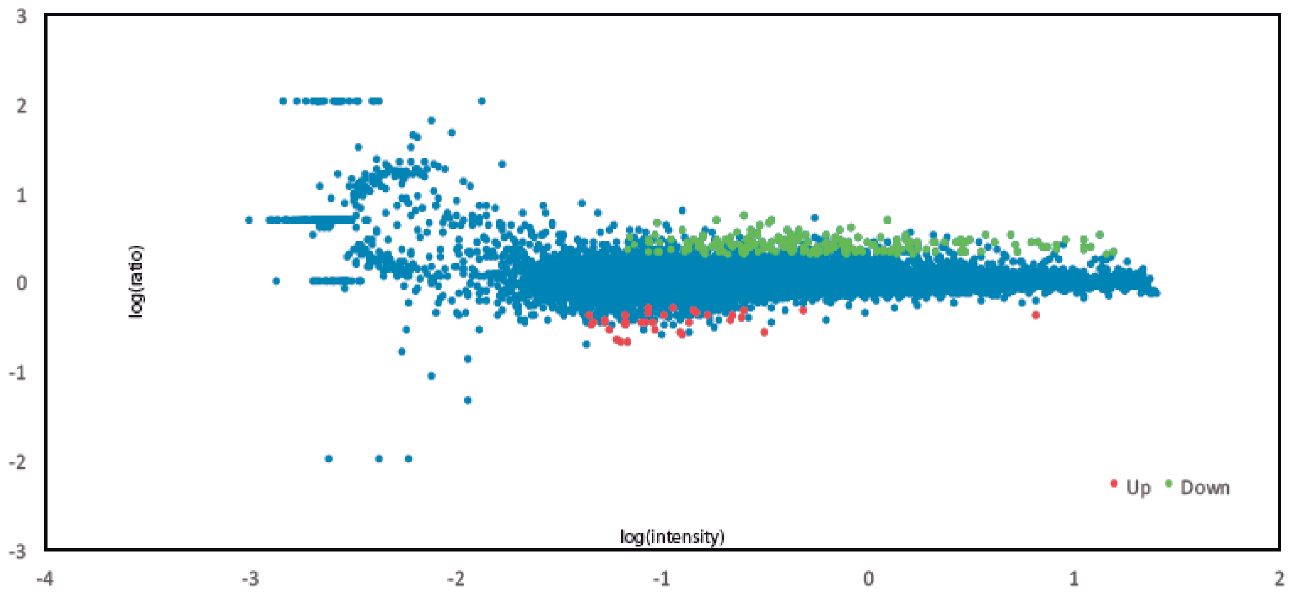

B

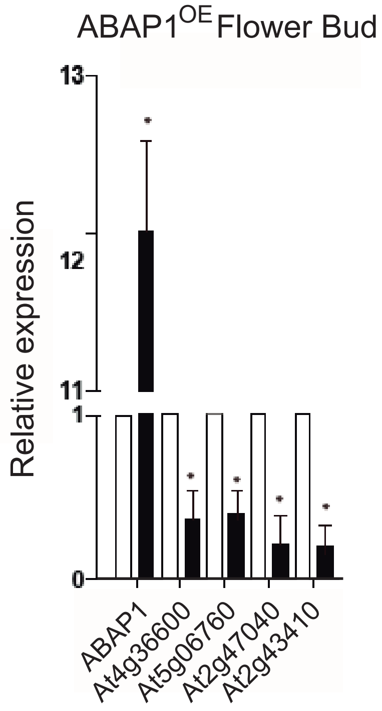

Figure S6 (refers to Figure 6): Differentially expressed genes in ABAP1^{OE} flowers, analyzed by microarray. **(A)** Ratio-Intensity (R-I) plot from experimental data of three independent biological replicates after normalization and replicate analysis. log(ratio)=log10 (WT/ABAP1^{OE}); log(intensity)=0.5 log10 (WT . ABAP1^{OE}), where WT and ABAP1^{OE} are signal intensities for a given element in a mutant or the wild-type, respectively. Genes upregulated in ABAP1^{OE} are shown in red and downregulated genes are shown in green. **(B)** Relative mRNA levels of genes downregulated in ABAP1^{OE} array were determined by qRT-PCR in wild-type and ABAP1^{OE} flower buds. Data were normalized with *UBQ14* as reference gene and were compared with wild-type. ABAP1 expression is shown as control. Each biological replicate was performed with a pool of 15 flower buds. Bars indicate mean ± standard error of biological replicates. A statistical analysis was performed by t-test (p-value <0.05). Asterisks (*) indicate significant changes.

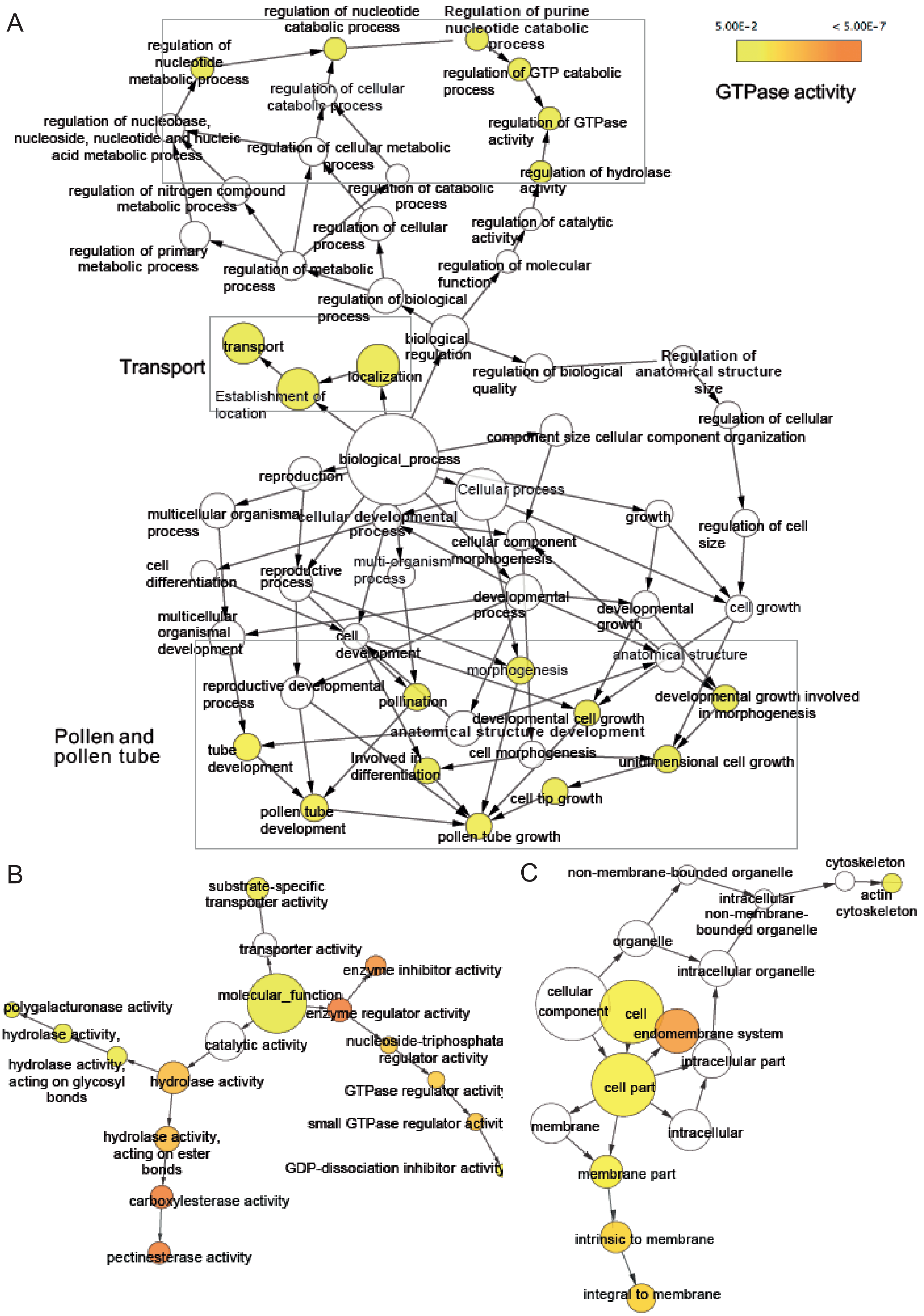

Figure S7 (refers to Figure 6): Gene Ontology (GO) term network analysis of down regulated genes in ABAP1^{OE} array based on biological processes, molecular function and cellular component. Functional enrichment analysis was performed for downregulated genes with the BINGO plug-in in Cytoscape. Assigned GO terms were used to classify functions of downregulated genes based on biological processes **(A),** molecular function **(B)** and cellular component **(C)**. The yellow and orange nodes represent terms with significant enrichment, with darker orange representing a higher degree of significance, as shown by the legend on graph. White nodes are terms with no significant enrichment, but are included because they have a significant child term.
